# Analysis on Optimal Error Exponents of Binary Classification for Source with Multiple Subclasses

**DOI:** 10.3390/e24050635

**Published:** 2022-04-30

**Authors:** Hiroto Kuramata, Hideki Yagi

**Affiliations:** Department of Computer and Network Engineering, The University of Electro-Communications, 1-5-1 Chofugaoka, Chofu 182-8585, Tokyo, Japan; h.kuramata@mail.uec.jp

**Keywords:** binary classification, error exponent, multiple subclasses

## Abstract

We consider a binary classification problem for a test sequence to determine from which source the sequence is generated. The system classifies the test sequence based on empirically observed (training) sequences obtained from unknown sources $P_{1}$ and $P_{2}$. We analyze the asymptotic fundamental limits of statistical classification for sources with multiple subclasses. We investigate the first- and second-order maximum error exponents under the constraint that the type-I error probability for all pairs of distributions decays exponentially fast and the type-II error probability is upper bounded by a small constant. In this paper, we first give a classifier which achieves the asymptotically maximum error exponent in the class of deterministic classifiers for sources with multiple subclasses, and then provide a characterization of the first-order error exponent. We next provide a characterization of the second-order error exponent in the case where only $P_{2}$ has multiple subclasses but $P_{1}$ does not. We generalize our results to classification in the case that $P_{1}$ and $P_{2}$ are a stationary and memoryless source and a mixed memoryless source with general mixture, respectively.

## 1. Introduction

### 1.1. Background

The problem of learning sources from training sequences and estimating the source from which a test sequence is generated is known as a classification problem. Recently, this problem has been actively studied in a field such as machine learning, and it is desirable to conduct studies that guarantee the performance of the system. In the field of information theory, studies have been conducted mainly to analyze the performance of optimal tests. When the number of sources is two, the binary classification problem can be regarded as a binary hypothesis testing problem, using training sequences. In the setting of binary hypothesis testing, it is assumed that the sources are known, but in real-world applications, the sources are not known in general. Therefore, it is of importance to consider the binary classification problem.

Hypothesis testing includes approaches such as the Bayesian test [1,2] and the Neyman–Pearson test [3,4,5]. In this paper, we take the latter approach to formulate the best asymptotic error exponent (the exponential part of an error probability).

There are a lot of studies related to the classification problem. We state important points, which are deeply connected to this study in some previous studies. Gutman [3] has shown that type-based (empirical distribution-based) tests asymptotically achieve the maximum type-II error exponent for stationary Markov sources, while the type-I error probability exponentially converges to zero as the length of a test sequence goes to infinity. Zhou et al. [5] derived second-order approximations of the maximum type-I error exponent for stationary and memoryless sources when the type-II error probability is upper bounded by a small constant. On the other hand, for the hypothesis testing problem, Han and Nomura [6] characterized a first-order maximum error exponent when each sequence is generated from a mixed memoryless source, which is a mixture of stationary and memoryless sources. In addition, they also characterized a second-order maximum error exponent in the case where one source is a stationary and memoryless source and the other source is a mixed memoryless source.

### 1.2. Contributions

In this paper, we investigate the binary classification problem for stationary memoryless sources with multiple subclasses. The class of sources with multiple subclasses is important in binary classification because there are many such settings in real-world applications. For example, newspaper articles with science headlines consist of topics of physics, chemistry, biology, etc. We assume that sources (subclasses) are characterized by a mixture with some unknown prior distribution (cf. Equation (2)), and the overall sources can be regarded as mixed memoryless sources [6]. The purpose of this paper is to characterize the first- and second-order maximum error exponents in a single-letter form (the term “single-letter form” means an expression which does not depend on lengths of sequences *n* or *N* (cf. the formulas for error exponents in Theorems 2–4)).

To this end, we generalize Gutman’s classifier [3], which was shown to be first- and second-order optimal for memoryless sources (with no multiple subclasses) in [5]. This classifier uses training sequences from one of the two sources, as in [3,4,5], making a type-based decision for a source (subclass) with the smallest skewed Jensen–Shannon divergence [7] among the subclasses. We show that this classifier asymptotically achieves the maximum type-II error exponent in the class of deterministic classifiers for a given pair of distributions when the type-I error probability decays exponentially fast for all pairs of distributions in Theorem 1. We also demonstrate that the structure of this classifier leads to a reversed and more relaxed relation; the maximization of the type-I error exponent when the type-II error probability is upper bounded by a small constant ϵ(0≤ϵ<1) for sources with multiple subclasses in Theorem 2. In addition, using the Berry–Esseen theorem [8], we derive the second-order maximum error exponent in the case where only one of sources has subclasses in Theorem 3. Finally, the fact that the classifier uses the test sequence from one of two sources motivates us to consider a more general case; the first source is a source with no multiple subclasses, but the second source is given by a general mixture [6,9]. That is, the number of subclasses is not necessarily finite, and the prior distribution of subclasses may not be discrete (cf. Equation (75)). We give characterizations of the first- and second-order maximum error exponents in Theorem 4.

### 1.3. Related Work

Ziv [10] proposed a classifier based on empirical entropy and discussed the relationship between binary classification and universal source coding. Hsu and Wang [4] characterized the maximum error exponent with mismatched empirically observed statistics. In their achievability proof, a generalization of Gutman’s classifier is also used. Kelly et al. [11] investigated binary classification with large alphabets. Unnikrishnan and Huang [12] investigated the type-I error probability of binary classification using the analysis of weak convergence. Generalizing the binary classification problem, He et al. [13] discussed the binary distribution detection problem, in which a different generalization of Gutman’s classifier is also discussed.

There are also studies which take a Bayesian approach. Merhav and Ziv [1] analyzed the weighted sum of type-I and type-II error probabilities, and subsequently, Saito and Matsushima [2,14] gave a different result via the analysis for the Bayes code.

### 1.4. Organization

The rest of this paper is organized as follows: In Section 2, we define the notation used in this paper and describe the details of the source and system model. Moreover, we state the problem setting, defining the first- and second-order maximum error exponents. In Section 3, we first give a classifier which achieves the asymptotically maximum error exponent in the class of deterministic classifiers for sources with multiple subclasses. Next, we characterize the first- and second-order maximum error exponents and the detailed proofs for the first-order representation. In Section 4, we generalize the obtained results to the classification of a mixed memoryless source with general mixture. In Section 5, we present numerical examples. Finally, in Section 6, we provide some concluding remarks and future work.

## 2. Problem Formulation

### 2.1. Notation

The set of non-negative real numbers is denoted by $R^{+}$. Calligraphic X stands for a finite alphabet. Upper-case *X* denotes a random variable taking values in X, and lower-case x∈X denotes its realization. Throughout this paper, logarithms are of base *e*. For integers *a* and *b* such that a≤b,[a,b] denotes the set {a,a+1,⋯,b}. The set of all probability distributions on a finite set X is denoted as P(X). Notation regarding the method of types [15] is as follows: Given a vector $x_{1}^{n}=\left(x_{1},x_{2},\cdots ,x_{n}\right)\in X^{n}$, the type is denoted as

(1) $$\begin{array}{c} q_{x_{1}^{n}}\left(a\right)=\frac{1}{n}\sum_{i=1}^{n}1\;\left\{x_{i}=a\right\},\;\;a\in X. \end{array}$$

The set of types formed from length-*n* sequences with alphabet X is denoted as $P_{n}\left(X\right)$. The probability that *n* independent drawings from a probability distribution Q∈P(X) give $x\in X^{n}$ is denoted by Q(x).

### 2.2. Source with Multiple Subclasses

Consider a source consisting of multiple subclasses. Each subclass is distributed according to a given probability (weight). Let ${\left\{P_{1}^{i}\right\}}_{i\in S}$ be a family of probability distributions on a finite alphabet X, where S={1,⋯,s} is a probability space with probability measure v(i),i∈S. That is, the probability of $x\in X^{n}$ is given by

(2) $$\begin{array}{c} P_{1}\left(x\right)=\sum_{i=1}^{s}v\left(i\right)P_{1}^{i}\left(x\right), \end{array}$$

where the *i*-th subclass $P_{1}^{i}$ is a stationary and memoryless source. That is, for $x=\left(x_{1},x_{2},\cdots ,x_{n}\right)\in X^{n}$,

(3) $$\begin{array}{c} P_{1}^{i}\left(x\right)=\prod_{j=1}^{n}P_{1}^{i}\left(x_{j}\right) \end{array}$$

(for notational simplicity, we denote both the multi-letter and single-letter probabilities by $P_{1}^{i}$ with a slight abuse of notation).

In view of (2), the sequence x can be regarded as an output from a mixed memoryless source $P_{1}\left(\cdot \right)$, and it is called a test sequence. Similarly, let ${\left\{P_{2}^{i}\right\}}_{i\in U}$ be a family of probability distributions, where U={1,⋯,u} is a probability space with probability measure w(i),i∈U. For these mixed sources, if the sources are known, that is, the addressed problem is hypothesis testing, the first- and second-order error exponents were analyzed by Han and Nomura [6]. In this paper, we assume that the sources are unknown and training sequences are available to learn about the source. Sets of training sequences are denoted by $t_{1}=\left\{t_{1}^{1},\cdots ,t_{1}^{s}\right\}$ and $t_{2}=\left\{t_{2}^{1},\cdots ,t_{2}^{u}\right\}$, where $t_{i}^{j}\in X^{N}$ of length *N* is output from subclass *j* and N=⌈nγ⌉ for some fixed $\gamma \in R^{+}$. Then, the joint probabilities of training sequences are, respectively,

(4) $$\begin{array}{c} P_{1}\left(t_{1}\right)=\prod_{i=1}^{s}P_{1}^{i}\left(t_{1}^{i}\right), \end{array}$$

(5) $$\begin{array}{c} P_{2}\left(t_{2}\right)=\prod_{j=1}^{u}P_{2}^{j}\left(t_{2}^{j}\right). \end{array}$$

We define the class of sources with multiple subclasses on a probability space S with probability measure v(·) as

(6) $$\begin{array}{c} P_{S}\left(X\right):=\left\{P={\left\{P^{i},v\left(i\right)\right\}}_{i\in S}:P^{i}\in P\left(X\right)\right\}, \end{array}$$

which means $P_{1}\in P_{S}\left(X\right)$, where the set of weights ${\left\{v\left(i\right)\right\}}_{i\in S}$ is implicitly fixed. Similarly, we define the class of sources with multiple subclasses on a probability space U with probability measure w(·) as

(7) $$\begin{array}{c} P_{U}\left(X\right):=\left\{P={\left\{P^{i},w\left(i\right)\right\}}_{i\in U}:P^{i}\in P\left(X\right)\right\}, \end{array}$$

which means $P_{2}\in P_{U}\left(X\right)$.

### 2.3. System Model

**Figure 1:**
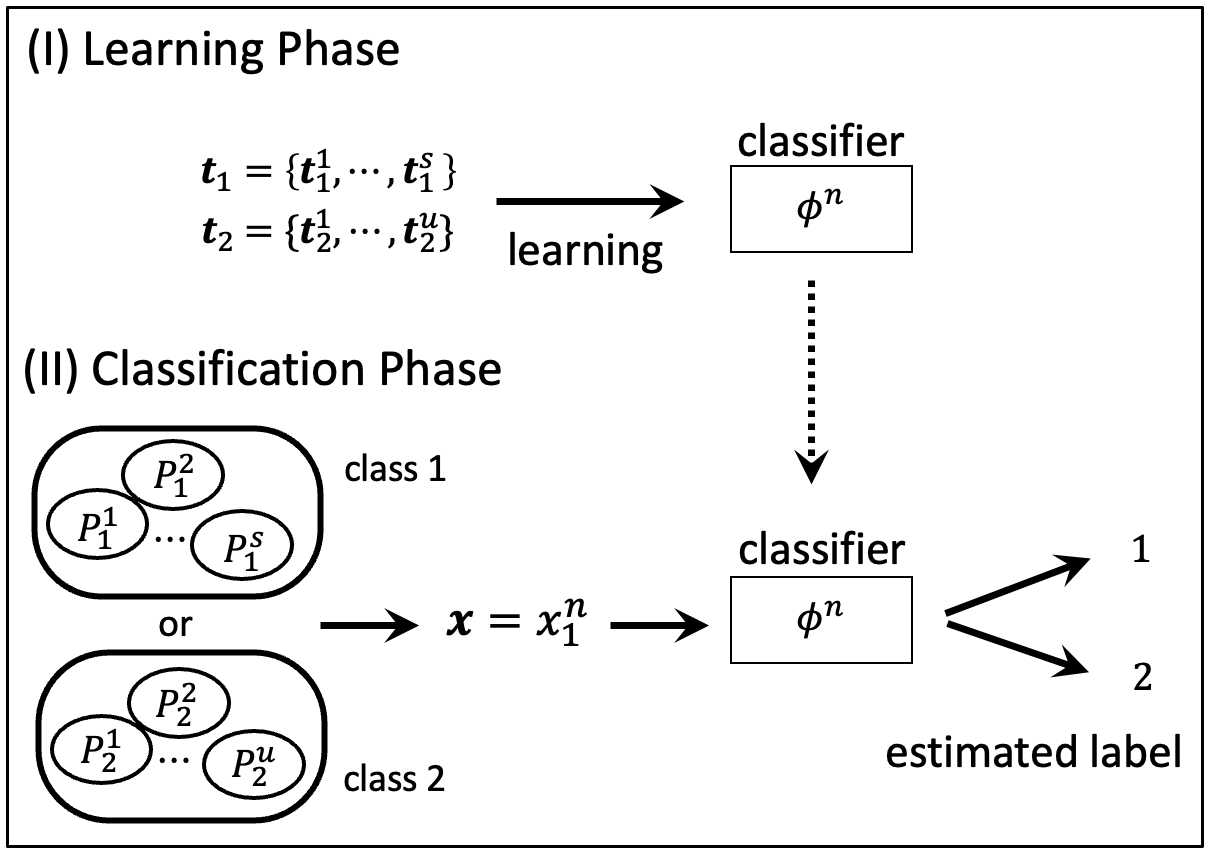
System model.

The binary classification problem assumed in this paper is shown in Figure 1. It consists of two phases: (I) learning phase and (II) classification phase. We explain the details of each phase.

(I) Learning phase: Determine the classifier by learning with the training sequences $t_{1}=\left\{t_{1}^{1},\cdots ,t_{1}^{s}\right\}$ and $t_{2}=\left\{t_{2}^{1},\cdots ,t_{2}^{u}\right\}$ generated from unknown source $P_{1}\in P_{S}\left(X\right)$ and $P_{2}\in P_{U}\left(X\right)$, respectively.

(II) Classification phase: It represents the phase in which we judge whether the test sequence $x\in X^{n}$ generated from $P_{1}\in P_{S}\left(X\right)$ or $P_{2}\in P_{U}\left(X\right)$ according to the classifier determined in (I).

### 2.4. Maximum Error Exponent

In this section, we define two error probabilities that arise in a binary classification problem and formulate the maximum error exponents. In the binary classification problem, a test is described as a partition of the space $\phi^{n}:X^{n}\times X^{(s+u)N}\to \left\{1,2\right\}$. The type-I and type-II error probabilities of a given test $\phi^{n}$ are denoted as $\beta_{1}\left(\phi^{n}|P_{1},P_{2}\right)$ and $\beta_{2}\left(\phi^{n}|P_{1},P_{2}\right)$, respectively. That is,

(8) $$\begin{array}{c} \beta_{1}\left(\phi^{n}|P_{1},P_{2}\right):=P_{1}\left\{\phi^{n}\left(x,t_{1},t_{2}\right)=2\right\}, \end{array}$$

(9) $$\begin{array}{c} \beta_{2}\left(\phi^{n}|P_{1},P_{2}\right):=P_{2}\left\{\phi^{n}\left(x,t_{1},t_{2}\right)=1\right\}. \end{array}$$

Here, $P_{\theta }\left\{\cdot \right\}$ is the joint probability of training and testing sequences when the underlying parameter is θ, given by

(10) $$\begin{array}{cc} \; & P_{1}\left\{\phi^{n}\left(x,t_{1},t_{2}\right)=\ell \right\}:=\sum_{\left(x,t_{1},t_{2}\right):\;\phi^{n}\left(x,t_{1},t_{2}\right)=\ell }P_{1}\left(x\right)\prod_{i=1}^{s}P_{1}^{i}\left(t_{1}^{i}\right)\prod_{j=1}^{u}P_{2}^{j}\left(t_{2}^{j}\right)\;\;\;\;\;\;\;\;\ell \in \left\{1,2\right\}, \end{array}$$

(11) $$\begin{array}{cc} \; & P_{2}\left\{\phi^{n}\left(x,t_{1},t_{2}\right)=\ell \right\}:=\sum_{\left(x,t_{1},t_{2}\right):\;\phi^{n}\left(x,t_{1},t_{2}\right)=\ell }P_{2}\left(x\right)\prod_{i=1}^{s}P_{1}^{i}\left(t_{1}^{i}\right)\prod_{j=1}^{u}P_{2}^{j}\left(t_{2}^{j}\right)\;\;\;\;\;\;\;\;\ell \in \left\{1,2\right\}. \end{array}$$

We consider the problem of maximizing the type-I error exponent when the type-II error probability is upper bounded by a small constant ϵ∈[0,1). In this study, we characterize the following quantities (the first- and second-order maximum type-I error exponent).

**Definition** **1** (First-order maximum error exponent). *For any pair of distributions $P=\left(P_{1},P_{2}\right)\in P_{S}\left(X\right)\times P_{U}\left(X\right)$ and ϵ∈[0,1), we define*

(12) $$\begin{array}{cc} \hat{\lambda }\left(\epsilon \right) & :=\sup\left\{\lambda \in R^{+}|\begin{array}{c} \exists {\left\{\phi^{n}\right\}}_{n=1}^{\infty }\;\;s.t.\;\mathrm{for}\;\mathrm{all}\;\mathrm{sufficiently}\;\mathrm{large}\;n\\ \beta_{1}\left(\phi^{n}|\tilde{P}\right)\leq \exp\left(-n\lambda \right)\;\;\left(\forall \tilde{P}\in P_{S}\left(X\right)\times P_{U}\left(X\right)\right),\\ \underset{n\to \infty }{lim sup}\beta_{2}\left(\phi^{n}|P\right)\leq \epsilon \end{array}\right\}, \end{array}$$

*where the weights of $\tilde{P}=\left({\tilde{P}}_{1},{\tilde{P}}_{2}\right)\in P_{S}\left(X\right)\times P_{U}\left(X\right)$ are the sames as the weights of $P=\left(P_{1},P_{2}\right)\in P_{S}\left(X\right)\times P_{U}\left(X\right)$.*

**Definition** **2** (Second-order maximum error exponent). *For any pair of distributions $P=\left(P_{1},P_{2}\right)\in P_{S}\left(X\right)\times P_{U}\left(X\right)$ and ϵ∈[0,1), we define*

(13) $$\begin{array}{c} \hat{r}\left(\epsilon ,\lambda \right):=\sup\left\{r\;|\begin{array}{c} \exists {\left\{\phi^{n}\right\}}_{n=1}^{\infty }\;\;s.t.\;\mathrm{for}\;\mathrm{all}\;\mathrm{sufficiently}\;\mathrm{large}\;n\\ \beta_{1}\left(\phi^{n}|\tilde{P}\right)\leq \exp\left(-n\lambda -\sqrt{n}r\right)\;\;\left(\forall \tilde{P}\in P_{S}\left(X\right)\times P_{U}\left(X\right)\right),\\ \underset{n\to \infty }{lim sup}\beta_{2}\left(\phi^{n}|P\right)\leq \epsilon \end{array}\right\}, \end{array}$$

*where the weights of $\tilde{P}=\left({\tilde{P}}_{1},{\tilde{P}}_{2}\right)\in P_{S}\left(X\right)\times P_{U}\left(X\right)$ are the sames as the weights of $P=\left(P_{1},P_{2}\right)\in P_{S}\left(X\right)\times P_{U}\left(X\right)$.*

**Remark** **1.** *In (12) and (13), the type-I error probability is constrained for any $\tilde{P}\in P_{S}\left(X\right)\times P_{U}\left(X\right)$ for technical reasons. In more detail, this condition is required in the proof of the converse part. This condition was also imposed by Gutman [3], Hsu and Wang [4], and Zhou et al. [5].*

In Definitions 1 and 2, we focus on universal tests that perform well for all pairs of distributions with respect to the type-I error probability, and at the same time, constrain the type-II error probability with respect to a particular pair of distributions. We obtain the same result when the weights of $\left({\tilde{P}}_{1},{\tilde{P}}_{2}\right)\in P_{S}\left(X\right)\times P_{U}\left(X\right)$ are not fixed.

## 3. Main Result

### 3.1. A Test to Achieve Maximum Error Exponent

The rule for estimating whether a test sequence generated from $P_{1}\in P_{S}\left(X\right)$ or $P_{2}\in P_{U}\left(X\right)$ is called a decision rule. One of the goals of the classification problem is to design an optimal decision rule which achieves a maximum error exponent based on training sequences. In this section, we present a decision rule that asymptotically achieves the maximum type-II error exponent for any pair of distributions when the type-I error exponent is lower bounded by a constant for all pairs of distributions (cf. Theorem 1).

To define a test that is asymptotically optimum, we define two generalizations of the Jensen–Shannon divergence. These generalizations are related to some variational definitions in [7,16]. For any pair of distributions $\left(Q_{1},Q_{2}\right)\in P{\left(X\right)}^{2}$ and any number $\gamma \in R^{+}$, let the *generalized Jensen–Shannon divergence* be

(14) $$\begin{array}{cc} \; & \mathrm{GJS}\left(Q_{1},Q_{2},\gamma \right):=\gamma D\left(Q_{1}\left|\right|\frac{\gamma Q_{1}+Q_{2}}{1+\gamma }\right)+D\left(Q_{2}\left|\right|\frac{\gamma Q_{1}+Q_{2}}{1+\gamma }\right), \end{array}$$

where D(p||q) denotes the Kullback–Leibler divergence for p∈P(X) and q∈P(X) defined as

(15) $$\begin{array}{c} D\left(p||q\right):=\sum_{i\;\in X}p\left(i\right)\log\frac{p(i)}{q(i)}. \end{array}$$

The generalized Jensen–Shannon divergence $\mathrm{GJS}(Q_{1},Q_{2},\gamma )$ corresponds to a skewed α-Jensen–Shannon divergence for $\alpha =\frac{\gamma }{1+\gamma }$. Additionally, for $\left(Q_{1},Q_{2}\right)\in P_{S}\left(X\right)\times P\left(X\right)$, we define the *minimized generalized Jensen–Shannon divergence* by

(16) $$\begin{array}{c} \mathrm{MGJS}\left(Q_{1},Q_{2},\gamma \right):=\min_{i\in S}\mathrm{GJS}\left(Q_{1}^{i},Q_{2},\gamma \right). \end{array}$$

Given a threshold $\lambda \in R^{+}$ (including λ=0), the decision rule to achieve the maximum error exponent is given by

(17) $$\begin{array}{c} \Lambda_{2}^{n}=\left\{\left(x,t_{1},t_{2}\right)\in X^{n}\times X^{sN}\times X^{uN}\;|\;\mathrm{MGJS}\left(q_{t_{1}},q_{x},\gamma \right)\geq \tilde{\lambda }\right\}, \end{array}$$

where $\tilde{\lambda }=\lambda +\eta \left(n\right)$, $\eta \left(n\right):=\frac{2\log s+|X|\log(n+N+1)}{n}$ and $\Lambda_{i}^{n},i\in \left\{1,2\right\}$ is the set of $\left(x,t_{1},t_{2}\right)$ determined to be class *i* by the test $\Lambda^{n}$. By definition, the discriminant function $\mathrm{MGJS}(q_{t_{1}},q_{x},\gamma )$, appearing on the right-hand side of (17), can also be expressed as

(18) $$\begin{array}{c} \mathrm{MGJS}\left(q_{t_{1}},q_{x},\gamma \right)\;=\;\min_{i\in S}\left\{\gamma D(q_{t_{1}}\left|\right|q_{y_{1}^{i}})+D(q_{x}\left|\right|q_{y_{1}^{i}})\right\}, \end{array}$$

where $y_{1}^{i}:=xt_{1}^{i}$. From (17) and (18), $\Lambda^{n}$ is a type-based test and implicitly depends on λ. In addition, this test uses training sequences asymmetrically; only sequence $t_{1}$ is used, but not $t_{2}$ (cf. refs. [3,4,5]).

**Theorem** **1.** *For any given $\lambda \in R^{+}$ and any sequence of tests $\left\{\phi^{n}\right\}$ such that $\beta_{1}\left(\phi^{n}|\tilde{P}\right)\leq \exp\left(-n\lambda \right),\forall \tilde{P}=\left({\tilde{P}}_{1},{\tilde{P}}_{2}\right)\in P_{S}\left(X\right)\times P_{U}\left(X\right)$, the sequence of tests $\left\{\Lambda^{n}\right\}$ given by (17) satisfies for any pair of distributions $P=\left(P_{1},P_{2}\right)\in P_{S}\left(X\right)\times P_{U}\left(X\right)$,*

(19) $$\begin{array}{cc} \beta_{1}\left(\Lambda^{n}|\tilde{P}\right) & \leq \exp\left(-n\lambda \right)\;\;\;(\forall \tilde{P}\in P_{S}\left(X\right)\times P_{U}\left(X\right)), \end{array}$$

(20) $$\begin{array}{cc} \lim_{n\to \infty }-\frac{1}{n}\log\beta_{2}\left(\phi^{n}|P\right) & \leq \lim_{n\to \infty }-\frac{1}{n}\log\beta_{2}\left(\Lambda^{n}|P\right). \end{array}$$

**Proof.**  Equation (19) is derived in Section 3.3.1. The proof of (20) follows from Corollary 1. Although there is a deviation between the exponents in Corollary 1 and for the test $\Lambda^{n}$, the deviation vanishes asymptotically. □

Theorem 1 shows that the test $\Lambda^{n}$ can asymptotically achieve the maximum type-II error exponent among the tests $\phi^{n}$ for which the type-I error exponent is greater than or equal to λ. This test also has a reversed and more relaxed property; it achieves $\hat{\lambda }\left(\epsilon \right)$, the maximum type-I error exponent when the type-II error probability is upper bounded by a constant ϵ(0≤ϵ<1) (see the achievability proof of Theorem 2 in Section 3.3.1).

### 3.2. First-Order Maximum Error Exponent

In this section, we characterize the first-order maximum error exponent in a single-letter form for sources with multiple subclasses.

**Theorem** **2.** *For any pair of distributions $\left(P_{1},P_{2}\right)\in P_{S}\left(X\right)\times P_{U}\left(X\right)$, we have*

(21) $$\begin{array}{c} \hat{\lambda }\left(\epsilon \right)=\sup\left\{\bar{\lambda }\;|\sum_{\left\{j\in U:\;\mathrm{MGJS}\left(P_{1},P_{2}^{j},\gamma \right)<\bar{\lambda }\right\}}w\left(j\right)\leq \epsilon \right\}. \end{array}$$

*It should be noted that $\hat{\lambda }\left(\epsilon \right)$ depends on ${\left\{w\left(j\right)\right\}}_{j\in U}$, but not on ${\left\{v\left(i\right)\right\}}_{i\in S}$.*

**Proof.**  The proof is provided in Section 3.3. □

**Remark** **2.** *If S and U are singletons (that is, s=1 and u=1), Theorem 2 reduces to the following formula given by Zhou et al. [5]:*

(22) $$\begin{array}{c} \hat{\lambda }\left(\epsilon \right)=\mathrm{GJS}\left(P_{1},P_{2},\gamma \right)\;\;\left(0\leq \forall \epsilon <1\right), \end{array}$$

*which means that $\hat{\lambda }\left(\epsilon \right)$ does not depend on ϵ and the strong converse holds in this case, unlike in the case |S|,|U|>1. On the other hand, for general S and U but in the spacial case of ϵ=0, formula (21) reduces to*

(23) $$\begin{array}{c} \hat{\lambda }\left(0\right)=\min_{i\in S,j\in U}\mathrm{GJS}\left(P_{1}^{i},P_{2}^{j},\gamma \right). \end{array}$$

### 3.3. Proof of Theorem 2

We divide the proof of Theorem 2 into two parts: the achievability (direct) part and the converse part.

#### 3.3.1. Achievability Part

In the achievability proof, we use the type-based test $\Lambda^{n}$ given by (17). Fix any

(24) $$\begin{array}{c} \lambda <\sup\left\{\bar{\lambda }\;|\sum_{\left\{j\in U:\;\mathrm{MGJS}\left(P_{1},P_{2}^{j},\gamma \right)<\bar{\lambda }\right\}}w\left(j\right)\leq \epsilon \right\}. \end{array}$$

Then, for any pair of distributions $\left(P_{1},P_{2}\right)\in P_{S}\left(X\right)\times P_{U}\left(X\right)$ and for all pairs of distributions $\left({\tilde{P}}_{1},{\tilde{P}}_{2}\right)\in P_{S}\left(X\right)\times P_{U}\left(X\right)$, we show

(25) $$\begin{array}{cc} \beta_{1}\left(\Lambda^{n}|{\tilde{P}}_{1},{\tilde{P}}_{2}\right) & \leq \exp(-n\lambda ), \end{array}$$

(26) $$\begin{array}{cc} \underset{n\to \infty }{lim sup}\beta_{2}\left(\Lambda^{n}|P_{1},P_{2}\right) & \leq \epsilon . \end{array}$$

First, we prove (25). For preliminaries, we define the following sets used in the proof:

(27) $$\begin{array}{cc} T_{n}\left(q_{x}\right) & :=\left\{x^{\prime }\in X^{n}:q_{x^{\prime }}=q_{x}\right\}, \end{array}$$

(28) $$\begin{array}{cc} {\tilde{\Lambda }}_{2}^{n} & :=\left\{\left(x,t_{1}\right):\mathrm{MGJS}\left(q_{t_{1}},q_{x},\gamma \right)\geq \tilde{\lambda }\right\}, \end{array}$$

(29) $$\begin{array}{cc} \Gamma (\Lambda ) & :=\left\{\left(q_{x},q_{t_{1}}\right):\left(x,t_{1}\right)\in \Lambda \right\}, \end{array}$$

where ${\tilde{\Lambda }}_{2}^{n}$ is the projection of $\Lambda_{2}^{n}\subseteq X^{n}\times X^{sN}\times X^{uN}$ onto the space $X^{n}\times X^{sN}$. To evaluate the probability of a source sequence being in $T_{n}\left(q_{x}\right)$, the following relationship holds from the *method of types* [15].

**Lemma** **1.** *Suppose that the sequence x is sampled independently from the source P∈P(X). Then,*

(30) $$\begin{array}{c} \frac{1}{|P_{n}\left(X\right)|}\exp\left\{-nD(q_{x}||P)\right\}\leq P\left(T_{n}\left(q_{x}\right)\right)\leq \exp\left\{-nD(q_{x}||P)\right\}, \end{array}$$

*where $P_{n}\left(X\right)$ denotes the set of types formed from length-n sequences with alphabet X and $|P_{n}{\left(X\right)|\leq \left(n+1\right)}^{\left|X\right|}$.*

Then, an upper bound on the type-II error probability of the test $\Lambda^{n}=\left(\Lambda_{1}^{n},\Lambda_{2}^{n}\right)$ for all pairs of distributions $\left({\tilde{P}}_{1},{\tilde{P}}_{2}\right)\in P_{S}\left(X\right)\times P_{U}\left(X\right)$ can be evaluated as follows:

$$\begin{array}{cc} \; & \beta_{1}\left(\Lambda^{n}|{\tilde{P}}_{1},{\tilde{P}}_{2}\right) \end{array}$$

(31) $$\begin{array}{c} =\sum_{\left(x,t_{1},t_{2}\right)\in \Lambda_{2}^{n}}{\tilde{P}}_{1}\left(x\right)\prod_{r=1}^{s}{\tilde{P}}_{1}^{r}\left(t_{1}^{r}\right)\prod_{j=1}^{u}{\tilde{P}}_{2}^{j}\left(t_{2}^{j}\right)\\ =\sum_{\left(x,t_{1}\right)\in {\tilde{\Lambda }}_{2}^{n}}\sum_{i=1}^{s}{\tilde{P}}_{1}^{i}\left(x\right)\prod_{r=1}^{s}{\tilde{P}}_{1}^{r}\left(t_{1}^{r}\right)v\left(i\right)\\ =\sum_{\left(q_{x},q_{t_{1}}\right)\in \Gamma \left(\Lambda_{2}^{n}\right)}\sum_{i=1}^{s}{\tilde{P}}_{1}^{i}\left(T_{n}\left(q_{x}\right)\right)\prod_{r=1}^{s}{\tilde{P}}_{1}^{r}\left(T_{N}\left(q_{t_{1}^{r}}\right)\right)v\left(i\right) \end{array}$$

(32) $$\begin{array}{cc} \; & \leq \sum_{\left(q_{x},q_{t_{1}}\right)\in \Gamma \left(\Lambda_{2}^{n}\right)}\sum_{i=1}^{s}\exp\left\{-nD(q_{x}\left|\right|{\tilde{P}}_{1}^{i})-\sum_{r=1}^{s}ND(q_{t_{1}^{r}}\left|\right|{\tilde{P}}_{1}^{r})\right\}\\ \; & \leq \sum_{\left(q_{x},q_{t_{1}}\right)\in \Gamma \left(\Lambda_{2}^{n}\right)}\sum_{i=1}^{s}\exp\left\{-nD(q_{x}\left|\right|{\tilde{P}}_{1}^{i})-ND(q_{t_{1}^{i}}\left|\right|{\tilde{P}}_{1}^{i})\right\} \end{array}$$

(33) $$\begin{array}{cc} \; & =\sum_{\left(q_{x},q_{t_{1}}\right)\in \Gamma \left(\Lambda_{2}^{n}\right)}\sum_{i=1}^{s}\exp\left\{-nD(q_{x}\left|\right|q_{y_{1}^{i}})-ND(q_{t_{1}^{i}}\left|\right|q_{y_{1}^{i}})-n\left(1+\gamma \right)D\left(\frac{q_{x}+\gamma q_{t_{1}^{i}}}{1+\gamma }\left|\right|{\tilde{P}}_{1}^{i}\right)\right\}, \end{array}$$

where (31) is derived from (4), (32) follows from Lemma 1 and (33) is derived in Appendix A. Minimizing exponents in (33) with respect to i∈S, we further obtain

(34) $$\begin{array}{cc} \; & \beta_{1}\left(\Lambda^{n}|{\tilde{P}}_{1},{\tilde{P}}_{2}\right)\\ \; & \leq \sum_{\left(q_{x},q_{t_{1}}\right)\in \Gamma \left(\Lambda_{2}^{n}\right)}s\exp\left\{-n\min_{i\in S}\left[D(q_{x}\left|\right|q_{y_{1}^{i}})+\gamma D(q_{t_{1}^{i}}\left|\right|q_{y_{1}^{i}})\right]\right\}\\ \; & \;\;\;\;\;\;\;\;\;\;\;\;\cdot \exp\left\{-n\min_{i\in S}\left(1+\gamma \right)D\left(\frac{q_{x}+\gamma q_{t_{1}^{i}}}{1+\gamma }\left|\right|{\tilde{P}}_{1}^{i}\right)\right\} \end{array}$$

(35) $$\begin{array}{cc} \; & \leq \sum_{\left(q_{x},q_{t_{1}}\right)\in \Gamma \left(\Lambda_{2}^{n}\right)}s\exp\left\{-n\tilde{\lambda }\right\}\cdot \exp\left\{-n\min_{i\in S}\;\left(1+\gamma \right)D\left(\frac{q_{x}+\gamma q_{t_{1}^{i}}}{1+\gamma }\left|\right|{\tilde{P}}_{1}^{i}\right)\right\}\\ \; & =\exp\left\{-n\left[\tilde{\lambda }-\frac{\log s}{n}\right]\right\}\cdot \sum_{\left(q_{x},q_{t_{1}}\right)\in \Gamma \left(\Lambda_{2}^{n}\right)}\exp\left\{-n\min_{i\in S}\;\left(1+\gamma \right)D\left(\frac{q_{x}+\gamma q_{t_{1}^{i}}}{1+\gamma }\left|\right|{\tilde{P}}_{1}^{i}\right)\right\}\\ \; & \leq \exp\left\{-n\left[\tilde{\lambda }-\frac{\log s}{n}\right]\right\}\cdot \sum_{Q\in P_{n+N}\left(X\right)}\exp\left\{\min_{i\in S}\;-\left(n+N\right)D(Q||{\tilde{P}}_{1}^{i})\right\} \end{array}$$

(36) $$\begin{array}{cc} \; & \leq \exp\left\{-n\left[\tilde{\lambda }-\frac{\log s}{n}\right]\right\}{\left(n+N+1\right)}^{\left|X\right|}\cdot \sum_{Q\in P_{n+N}\left(X\right)}\max_{i\in S}{\tilde{P}}_{1}^{i}\left(T_{n+N}\left(Q\right)\right)\\ \; & \leq \exp\left\{-n\left[\tilde{\lambda }-\frac{\log s}{n}\right]\right\}{\left(n+N+1\right)}^{\left|X\right|}\cdot \sum_{Q\in P_{n+N}\left(X\right)}\sum_{i=1}^{s}{\tilde{P}}_{1}^{i}\left(T_{n+N}\left(Q\right)\right)\\ \; & =\exp\left\{-n\left[\tilde{\lambda }-\frac{\log s}{n}\right]\right\}{\left(n+N+1\right)}^{\left|X\right|}\cdot \sum_{i=1}^{s}\sum_{Q\in P_{n+N}\left(X\right)}{\tilde{P}}_{1}^{i}\left(T_{n+N}\left(Q\right)\right)\\ \; & =\exp\left\{-n\left[\tilde{\lambda }-\frac{\log s}{n}\right]\right\}{\left(n+N+1\right)}^{\left|X\right|}s\\ \; & =\exp\left\{-n\left[\tilde{\lambda }-\frac{2\log s}{n}-\frac{\left|X|\log(n+N+1\right)}{n}\right]\right\}\\ \; & \;=\;\exp\left\{-n\lambda \right\}, \end{array}$$

where (34) is derived from (16) and (28), and (35) follows from Lemma 1.

Next, we demonstrate (26). For preliminaries, we define a new typical set and show some properties for the proof. For any given Q∈P(X), define the following typical set:

(37) $$\begin{array}{c} B_{n}\left(Q\right):=\left\{x\in X^{n}:\max_{x\in X}\left|q_{x}\left(x\right)-Q\left(x\right)\right|\leq \sqrt{\frac{\log n}{n}}\right\}. \end{array}$$

By using [6, Lemma 22], for $t_{1}∼Q_{1}$, $x∼Q_{2}$, generated from memoryless sources $\left(Q_{1},Q_{2}\right)\in P{\left(X\right)}^{2}$, we have

(38) $$\begin{array}{c} \mathrm{Pr}\left\{t_{1}\notin B_{N}\left(Q_{1}\right)\;\mathrm{or}\;x\notin B_{n}\left(Q_{2}\right)\right\}\leq \frac{\left(1+\gamma^{2})|X\right|}{\gamma^{2}n^{2}}. \end{array}$$

For any given x∈X and any pair of distributions $\left(Q_{1},Q_{2}\right)\in P{\left(X\right)}^{2}$, we define information density as

(39) $$\begin{array}{c} \iota_{i}\left(x|Q_{1},Q_{2},\gamma \right):=\log\frac{\left(1+\gamma \right)Q_{i}\left(x\right)}{\gamma Q_{1}\left(x\right)+Q_{2}\left(x\right)}\;\;\;\;\;\;\;\;\left(i=1,2\right). \end{array}$$

Furthermore, for any pair of distributions $\left(Q_{1},Q_{2}\right)\in P_{S}\left(X\right)\times P\left(X\right)$, define the function $i^{*}\left(Q_{1},Q_{2}\right)$, as the index of the subclass of $Q_{1}$, as follows:

(40) $$\begin{array}{cc} i^{*}\left(Q_{1},Q_{2}\right) & :=\underset{i\in S}{arg min}\left\{\gamma D\left(Q_{1}^{i}\left|\right|\frac{\gamma Q_{1}^{i}+Q_{2}}{1+\gamma }\right)+D\left(Q_{2}\left|\right|\frac{\gamma Q_{1}^{i}+Q_{2}}{1+\gamma }\right)\right\}\\ \; & \;=\underset{i\in S}{arg min}\mathrm{GJS}\left(Q_{1}^{i},Q_{2},\gamma \right). \end{array}$$

Hereafter, we denote $i^{*}\left(Q_{1},Q_{2}\right)$ simply as $i^{*}\left(Q_{2}\right)$ when the first argument is clear from the context.

**Lemma** **2.** *Assume that $\left(Q_{1},Q_{2}\right)\in P_{S}\left(X\right)\times P\left(X\right)$. For $q_{t_{1}^{i}}\in B_{N}\left(Q_{1}^{i}\right)\;\;\left(i\in S\right)$, $q_{x}\in B_{n}\left(Q_{2}\right)$, we have*

(41) $$\begin{array}{c} i^{*}\left(q_{t_{1}},q_{x}\right)\to i^{*}\left(Q_{1},Q_{2}\right)\;\;\;\;\;\;\left(n\to \infty \right). \end{array}$$

**Proof.**  The proof is provided in Appendix B. □

**Lemma** **3** (Zhou et al. [5]). *Assume that $\left(Q_{1},Q_{2}\right)\in P_{S}\left(X\right)\times P\left(X\right)$. For $q_{t_{1}^{i}}\in B_{N}\left(Q_{1}^{i}\right)\;\;\left(i\in S\right)$, $q_{x}\in B_{n}\left(Q_{2}\right)$, by applying the Taylor expansion to $\mathrm{GJS}(q_{t_{1}^{i}},q_{x},\gamma )$ around $\left(Q_{1}^{i},Q_{2}\right)$, we have*

(42) $$\begin{array}{c} \mathrm{GJS}\left(q_{t_{1}^{i}},q_{x},\gamma \right)=\frac{\gamma }{N}\sum_{m=1}^{N}\iota_{1}\left(t_{1,m}^{i}|Q_{1}^{i},Q_{2},\gamma \right)+\frac{1}{n}\sum_{m=1}^{n}\iota_{2}\left(x_{m}|Q_{1}^{i},Q_{2},\gamma \right)+O\left(\frac{\log n}{n}\right), \end{array}$$

*where $t_{1,m}^{i}$ denotes the m-th symbol of sequence $t_{1}^{i}$ and $x_{m}$ denotes the m-th symbol of sequence x.*

Note that the probability $P_{2}$ is calculated by assuming that the test sequence x is generated from $P_{2}$ (cf. Equation (11)). An upper bound on the type-II error probability can be evaluated as follows:

(43) $$\begin{array}{cc} \; & \underset{n\to \infty }{lim sup}\beta_{2}\left(\Lambda^{n}|P_{1},P_{2}\right)\\ \; & =\underset{n\to \infty }{lim sup}P_{2}\left\{\mathrm{MGJS}\left(q_{t_{1}},q_{x},\gamma \right)<\lambda +\eta \left(n\right)\right\}\\ \; & =\underset{n\to \infty }{lim sup}\sum_{j=1}^{u}w\left(j\right)P_{2}^{j}\left\{\mathrm{MGJS}\left(q_{t_{1}},q_{x},\gamma \right)<\lambda +\eta \left(n\right)\right\}\\ \; & \leq \underset{n\to \infty }{lim sup}\sum_{j=1}^{u}w\left(j\right)P_{2}^{j}\left\{\mathrm{MGJS}\left(q_{t_{1}},q_{x},\gamma \right)<\lambda +\eta \left(n\right),x\in B_{n}\left(P_{2}^{j}\right),t_{1}^{i}\in B_{N}\left(P_{1}^{i}\right),\forall i\in S\right\}\\ \; & \;\;\;\;\;\;\;\;\;\;\;\;\;\;\;+\underset{n\to \infty }{lim sup}\sum_{i=1}^{s}\sum_{j=1}^{u}w\left(j\right)\cdot P_{2}^{j}\left\{x\notin B_{n}\left(P_{2}^{j}\right)\;\mathrm{or}\;t_{1}^{i}\notin B_{N}\left(P_{1}^{i}\right)\right\}\\ \; & \leq \underset{n\to \infty }{lim sup}\sum_{j=1}^{u}w\left(j\right)P_{2}^{j}\left\{\mathrm{GJS}\left(q_{t_{1}^{i^{*}\left(q_{x}\right)}},q_{x},\gamma \right)<\lambda +\eta \left(n\right),x\in B_{n}\left(P_{2}^{j}\right),t_{1}^{i}\in B_{N}\left(P_{1}^{i}\right),\forall i\in S\right\}, \end{array}$$

where Equation (43) follows from Equations (14), (38) and (40). By using Lemma 3, Equation (43) can be expanded as follows:

$$\begin{array}{cc} \; & \underset{n\to \infty }{lim sup}\beta_{2}\left(\Lambda^{n}|P_{1},P_{2}\right)\\ \; & \leq \underset{n\to \infty }{lim sup}\sum_{j=1}^{u}w\left(j\right)P_{2}^{j}\{\frac{\gamma }{N}\sum_{m=1}^{N}\iota_{1}\left(t_{1,m}^{i^{*}\left(q_{x}\right)}|P_{1}^{i^{*}\left(q_{x}\right)},P_{2}^{j},\gamma \right)+\frac{1}{n}\sum_{m=1}^{n}\iota_{2}\left(x_{m}|P_{1}^{i^{*}\left(q_{x}\right)},P_{2}^{j},\gamma \right)<\lambda \\ & \;\;\;\;\;\;\;\;\;\;\;\;\;\;\;+O\left(\frac{\log n}{n}\right),x\in B_{n}\left(P_{2}^{j}\right),t_{1}^{i}\in B_{N}\left(P_{1}^{i}\right),\forall i\in S\}\\ \; & \leq \sum_{j=1}^{u}\underset{n\to \infty }{lim sup}w\left(j\right)P_{2}^{j}\{\frac{\gamma }{N}\sum_{m=1}^{N}\iota_{1}\left(t_{1,m}^{i^{*}\left(q_{x}\right)}|P_{1}^{i^{*}\left(q_{x}\right)},P_{2}^{j},\gamma \right)+\frac{1}{n}\sum_{m=1}^{n}\iota_{2}\left(x_{m}|P_{1}^{i^{*}\left(q_{x}\right)},P_{2}^{j},\gamma \right)<\lambda \end{array}$$

(44) $$\begin{array}{c} \;\;\;\;\;\;\;\;\;\;\;\;\;\;\;+O\left(\frac{\log n}{n}\right),x\in B_{n}\left(P_{2}^{j}\right),t_{1}^{i}\in B_{N}\left(P_{1}^{i}\right),\forall i\in S\}\\ =\sum_{j=1}^{u}\underset{n\to \infty }{lim sup}w\left(j\right)P_{2}^{j}\left\{x\in B_{n}\left(P_{2}^{j}\right),t_{1}^{i}\in B_{N}\left(P_{1}^{i}\right),\forall i\in S\right\}\\ \;\;\;\;\;\;\;\;\;\;\;\;\;\;\;\cdot P_{2}^{j}\{\frac{\gamma }{N}\sum_{m=1}^{N}\iota_{1}\left(t_{1,m}^{i^{*}\left(q_{x}\right)}|P_{1}^{i^{*}\left(q_{x}\right)},P_{2}^{j},\gamma \right)+\frac{1}{n}\sum_{m=1}^{n}\iota_{2}\left(x_{m}|P_{1}^{i^{*}\left(q_{x}\right)},P_{2}^{j},\gamma \right)<\lambda \end{array}$$

(45) $$\begin{array}{cc} \; & \;\;\;\;\;\;\;\;\;\;\;\;\;\;\;+O\left(\frac{\log n}{n}\right)\;\left|\;x\in B_{n}\left(P_{2}^{j}\right),t_{1}^{i}\in B_{N}\left(P_{1}^{i}\right),\forall i\in S\right\}, \end{array}$$

where Equation (44) is derived from Fatou’s lemma. It follows from (38) that

(46) $$\begin{array}{c} P_{2}^{j}\left\{t_{1}^{i}\in B_{N}\left(P_{1}^{i}\right),x\in B_{n}\left(P_{2}^{j}\right),\forall i\in S\right\}\to 1\;\;\;\;\left(n\to \infty \right). \end{array}$$

Here, Equation (14) can be also expressed as follows:

(47) $$\begin{array}{cc} \mathrm{GJS}(Q_{1},Q_{2},\gamma ) & =\gamma E_{Q_{1}}\left[\iota_{1}\left(X|Q_{1},Q_{2},\gamma \right)\right]+E_{Q_{2}}\left[\iota_{2}\left(X|Q_{1},Q_{2},\gamma \right)\right]. \end{array}$$

Therefore, by the weak law of large numbers, for any given δ>0,

(48) $$\begin{array}{cc} \; & \underset{n\to \infty }{lim sup}\mathrm{Pr}\{|\frac{\gamma }{N}\sum_{m=1}^{N}\iota_{1}\left(t_{1,m}^{i^{*}\left(q_{x}\right)}|P_{1}^{i^{*}\left(q_{x}\right)},P_{2}^{j},\gamma \right)+\frac{1}{n}\sum_{m=1}^{n}\iota_{2}\left(x_{m}|P_{1}^{i^{*}\left(q_{x}\right)},P_{2}^{j},\gamma \right)\\ \; & \;\;\;\;\;\;\;\;\;\;\;\;\;\;\;\;\;\;\;\;\;\;\;\;-\mathrm{GJS}(P_{1}^{i^{*}\left(q_{x}\right)},P_{2}^{j},\gamma )|\leq \delta \}=1. \end{array}$$

From this result of the weak law of large numbers and Lemma 2, combining (45)–(48) gives

(49) $$\begin{array}{cc} \underset{n\to \infty }{lim sup}\beta_{2}\left(\Lambda^{n}|P_{1},P_{2}\right)\; & \leq \sum_{\left\{j\in U:\;\mathrm{GJS}\left(P_{1}^{i^{*}\left(P_{2}^{j}\right)},P_{2}^{j},\gamma \right)<\lambda \right\}}w\left(j\right). \end{array}$$

Thus, by (24), we can see that

(50) $$\begin{array}{c} \underset{n\to \infty }{lim sup}\beta_{2}\left(\Lambda^{n}|P_{1},P_{2}\right)\leq \epsilon . \end{array}$$

#### 3.3.2. Converse Part

For any pair of distributions $\left(P_{1},P_{2}\right)\in P_{S}\left(X\right)\times P_{U}\left(X\right)$ and for all pairs of distributions $\left({\tilde{P}}_{1},{\tilde{P}}_{2}\right)\in P_{S}\left(X\right)\times P_{U}\left(X\right)$, fix any test $\phi^{n}$ satisfying that

(51) $$\begin{array}{cc} \beta_{1}\left(\phi^{n}|{\tilde{P}}_{1},{\tilde{P}}_{2}\right) & \leq \exp(-n\lambda ), \end{array}$$

(52) $$\begin{array}{cc} \underset{n\to \infty }{lim sup}\beta_{2}\left(\phi^{n}|P_{1},P_{2}\right) & \leq \epsilon . \end{array}$$

We show

(53) $$\begin{array}{c} \lambda \leq \sup\left\{\bar{\lambda }\;|\sum_{\left\{j\in U:\;\mathrm{MGJS}\left(P_{1},P_{2}^{j},\gamma \right)<\bar{\lambda }\right\}}w\left(j\right)\leq \epsilon \right\}. \end{array}$$

We first give some lemmas, which are useful in the proof of the converse part.

**Lemma** **4.** *Let $\phi^{n}$ be a test in which the decision rule depends only on $\left(x,t_{1},t_{2}\right)\in X^{n}\times X^{sN}\times X^{uN}$. Then, for any given κ∈[0,1], we can construct a type-based test $\Omega^{n}$ satisfying*

(54) $$\begin{array}{cc} \beta_{1}\left(\phi^{n}|{\tilde{P}}_{1},{\tilde{P}}_{2}\right) & \geq \kappa \beta_{1}\left(\Omega^{n}|{\tilde{P}}_{1},{\tilde{P}}_{2}\right), \end{array}$$

(55) $$\begin{array}{cc} \beta_{2}\left(\phi^{n}|{\tilde{P}}_{1},{\tilde{P}}_{2}\right) & \geq \left(1-\kappa \right)\beta_{2}\left(\Omega^{n}|{\tilde{P}}_{1},{\tilde{P}}_{2}\right) \end{array}$$

*for any pair of distributions $\left({\tilde{P}}_{1},{\tilde{P}}_{2}\right)\in P_{S}\left(X\right)\times P_{U}\left(X\right)$.*

**Proof.**  Lemma 4 can be proved in the same way as (Lemma 7 [5]), the proof of which is inspired by (Lemma 2 [3]). □

**Remark** **3.** *As in the proof of (Lemma 2 [3]) and (Lemma 7 [5]), a type-based test $\Omega^{n}$ specified in Lemma 4 is obtained by tailoring $\phi^{n}$ and satisfies Equations (54) and (55) for all $\left({\tilde{P}}_{1},{\tilde{P}}_{2}\right)\in P_{S}\left(X\right)\times P_{U}\left(X\right)$. In other words, the construction of $\Omega^{n}$ is universal, which is in the same spirit of (Lemma 2 [3]). This claim is slightly stronger than the one in (Lemma 7 [5]).*

**Lemma** **5.** *For any $\lambda \in R^{+}$, any type-based test $\Omega^{n}$ satisfying the condition that for all pairs of distributions $\left({\tilde{P}}_{1},{\tilde{P}}_{2}\right)\in P_{S}\left(X\right)\times P_{U}\left(X\right)$,*

(56) $$\begin{array}{c} \beta_{1}\left(\Omega^{n}|{\tilde{P}}_{1},{\tilde{P}}_{2}\right)\leq \exp\left(-n\lambda \right), \end{array}$$

*we have that for any pair of distributions $\left(P_{1},P_{2}\right)\in P_{S}\left(X\right)\times P_{U}\left(X\right)$*

(57) $$\begin{array}{c} \beta_{2}\left(\Omega^{n}|P_{1},P_{2}\right)\geq P_{2}\left\{\mathrm{MGJS}\left(q_{t_{1}},q_{x},\gamma \right)<\lambda -\rho \left(n\right)\right\}, \end{array}$$

*where $\rho \left(n\right):=\frac{|X|\log\left(n+1\right)+\left(s+u\right)|X|\log\left(N+1\right)-\log v^{*}}{n}$ with $v^{*}:=\min\left\{v\left(i\right):v\left(i\right)>0,i\in S\right\}$.*

**Proof.**  The proof is provided in Appendix C. □

The type-based test $\Omega^{n}$ specified in Lemma 4 satisfies Equations (54) and (55) for all $\left({\tilde{P}}_{1},{\tilde{P}}_{2}\right)\in P_{S}\left(X\right)\times P_{U}\left(X\right)$. If we set κ=1/n in Lemma 4, and combine it with Lemma 5, we can derive the following relation:

**Corollary** **1.** *For any given $\lambda \in R^{+}$, any test $\phi^{n}$ satisfying the condition that for all pairs of distributions $\left({\tilde{P}}_{1},{\tilde{P}}_{2}\right)\in P_{S}\left(X\right)\times P_{U}\left(X\right)$*

(58) $$\begin{array}{c} \beta_{1}\left(\phi^{n}|{\tilde{P}}_{1},{\tilde{P}}_{2}\right)\leq \exp\left(-n\lambda \right), \end{array}$$

*we have that for any pair of distributions $\left(P_{1},P_{2}\right)\in P_{S}\left(X\right)\times P_{U}\left(X\right)$*

(59) $$\begin{array}{cc} \; & \beta_{2}\left(\phi^{n}|P_{1},P_{2}\right)\geq \left(1-\frac{1}{n}\right)\cdot P_{2}\left\{\mathrm{MGJS}\left(q_{t_{1}},q_{x},\gamma \right)+\rho \left(n\right)+\frac{\log n}{n}<\lambda \right\}. \end{array}$$

**Proof.**  The proof is provided in Appendix D. □

By (59), a lower bound on the type-II error probability can be evaluated as follows:

(60) $$\begin{array}{cc} \; & \underset{n\to \infty }{lim sup}\beta_{2}\left(\phi^{n}|P_{1},P_{2}\right)\\ \; & \geq \underset{n\to \infty }{lim sup}\left(1-\frac{1}{n}\right)P_{2}\left\{\mathrm{MGJS}\left(q_{t_{1}},q_{x},\gamma \right)+O\left(\frac{\log n}{n}\right)<\lambda \right\}\\ \; & =\underset{n\to \infty }{lim sup}\sum_{j=1}^{u}w\left(j\right)P_{2}^{j}\left\{\mathrm{MGJS}\left(q_{t_{1}},q_{x},\gamma \right)+O\left(\frac{\log n}{n}\right)<\lambda \right\}\\ \; & \geq \underset{n\to \infty }{lim sup}\sum_{j=1}^{u}w\left(j\right)P_{2}^{j}\left\{\mathrm{MGJS}\left(q_{t_{1}},q_{x},\gamma \right)+O\left(\frac{\log n}{n}\right)<\lambda ,x\in B_{n}\left(P_{2}^{j}\right),t_{1}^{i}\in B_{N}\left(P_{1}^{i}\right),\forall i\in S\right\}\\ \; & =\underset{n\to \infty }{lim sup}\sum_{j=1}^{u}w\left(j\right)\cdot P_{2}^{j}\{\frac{1}{n}\sum_{m=1}^{N}\iota_{1}\left(t_{1,m}^{i^{*}\left(q_{x}\right)}|P_{1}^{i^{*}\left(q_{x}\right)},P_{2}^{j},\gamma \right)+\frac{1}{n}\sum_{m=1}^{n}\iota_{2}\left(x_{m}|P_{1}^{i^{*}\left(q_{x}\right)},P_{2}^{j},\gamma \right) \end{array}$$

(61) $$\begin{array}{cc} \; & \;\;\;\;\;\;\;\;\;\;\;\;\;\;\;+O\left(\frac{\log n}{n}\right)<\lambda ,x\in B_{n}\left(P_{2}^{j}\right),t_{1}^{i}\in B_{N}\left(P_{1}^{i}\right),\forall i\in S\}\\ \; & \geq \sum_{j=1}^{u}\underset{n\to \infty }{lim inf}w\left(j\right)\cdot P_{2}^{j}\{\frac{1}{n}\sum_{m=1}^{N}\iota_{1}\left(t_{1,m}^{i^{*}\left(q_{x}\right)}|P_{1}^{i^{*}\left(q_{x}\right)},P_{2}^{j},\gamma \right)+\frac{1}{n}\sum_{m=1}^{n}\iota_{2}\left(x_{m}|P_{1}^{i^{*}\left(q_{x}\right)},P_{2}^{j},\gamma \right) \end{array}$$

(62) $$\begin{array}{cc} \; & \;\;\;\;\;\;\;\;\;\;\;\;\;\;\;+O\left(\frac{\log n}{n}\right)<\lambda ,x\in B_{n}\left(P_{2}^{j}\right),t_{1}^{i}\in B_{N}\left(P_{1}^{i}\right),\forall i\in S\}\\ \; & \geq \sum_{j=1}^{u}\underset{n\to \infty }{lim inf}w\left(j\right)P_{2}^{j}\left\{x\in B_{n}\left(P_{2}^{j}\right),t_{1}^{i}\in B_{N}\left(P_{1}^{i}\right),\forall i\in S\right\}\\ \; & \;\;\;\;\;\;\;\;\;\;\;\;\;\;\;\cdot P_{2}^{j}\{\frac{\gamma }{N}\sum_{m=1}^{N}\iota_{1}\left(t_{1,m}^{i^{*}\left(q_{x}\right)}|P_{1}^{i^{*}\left(q_{x}\right)},P_{2}^{j},\gamma \right)+\frac{1}{n}\sum_{m=1}^{n}\iota_{2}\left(x_{m}|P_{1}^{i^{*}\left(q_{x}\right)},P_{2}^{j},\gamma \right)<\lambda \\ \; & \;\;\;\;\;\;\;\;\;\;\;\;\;\;\;+O\left(\frac{\log n}{n}\right)\;\left|\;x\in B_{n}\left(P_{2}^{j}\right),t_{1}^{i}\in B_{N}\left(P_{1}^{i}\right),\forall i\in S\right\}\\ \; & =\sum_{\left\{j\in U:\;\mathrm{GJS}\left(P_{1}^{i^{*}\left(P_{2}^{j}\right)},P_{2}^{j},\gamma \right)<\lambda \right\}}w\left(j\right), \end{array}$$

where Equations (60) and (61) are derived from Lemma 3 and Fatou’s lemma, respectively. Equation (62) follows from Equations (45)–(48). By Equation (52), Equation (62) indicates that

(63) $$\begin{array}{c} \lambda \leq \sup\left\{\bar{\lambda }\;\;|\sum_{\left\{j\in U:\;\mathrm{GJS}\left(P_{1}^{i^{*}\left(P_{2}^{j}\right)},P_{2}^{j},\gamma \right)<\bar{\lambda }\right\}}w\left(j\right)\leq \epsilon \right\}. \end{array}$$

### 3.4. Second-Order Maximum Error Exponent

In this section, we characterize the second-order maximum error exponent. For simplicity, we assume that only $P_{2}$ has subclasses, but $P_{1}$ does not (s=1). First, from Theorem 2 with s=1, the first-order maximum error exponent in this setting is characterized as follows: for any pair of distributions $\left(P_{1},P_{2}\right)\in P\left(X\right)\times P_{U}\left(X\right)$, we have

(64) $$\begin{array}{c} \hat{\lambda }\left(\epsilon \right)=\sup\left\{\bar{\lambda }\;|\sum_{\left\{j\in U:\;\mathrm{GJS}\left(P_{1},P_{2}^{j},\gamma \right)<\bar{\lambda }\right\}}w\left(j\right)\leq \epsilon \right\}, \end{array}$$

where $\mathrm{MGJS}(P_{1},P_{2}^{j},\gamma )$ in (21) is replaced by $\mathrm{GJS}(P_{1},P_{2}^{j},\gamma )$.

Next, we provide a characterization of the second-order maximum error exponent in Definition 2 with s=1 in the case where only $P_{2}$ has subclasses. By definition, $\hat{r}\left(\epsilon ,\lambda \right)=+\infty$ if $\lambda <\hat{\lambda }\left(\epsilon \right)$ and $\hat{r}\left(\epsilon ,\lambda \right)=-\infty$ if $\lambda >\hat{\lambda }\left(\epsilon \right)$. Therefore, in the discussion of the second-order error exponent, we focus on the case $\lambda =\hat{\lambda }\left(\epsilon \right)$.

**Theorem** **3.** *For any pair of distributions $\left(P_{1},P_{2}\right)\in P\left(X\right)\times P_{U}\left(X\right)$ and ϵ∈[0,1),*

(65) $$\begin{array}{cc} \hat{r}\left(\epsilon ,\lambda \right) & =\sup\left\{r\;|\sum_{\left\{j\in U:\;\mathrm{GJS}(P_{1},P_{2}^{j},\gamma )<\lambda \right\}}w\left(j\right)+\sum_{\left\{j\in U:\;\mathrm{GJS}(P_{1},P_{2}^{j},\gamma )=\lambda \right\}}\Phi_{j}\left(r\right)w\left(j\right)\leq \epsilon \right\}, \end{array}$$

*where $\lambda =\hat{\lambda }\left(\epsilon \right)$,*

(66) $$\begin{array}{cc} \Phi_{j}\left(r\right) & :=G\left(\frac{r}{\sqrt{V(P_{1},P_{2}^{j},\gamma )}}\right), \end{array}$$

(67) $$\begin{array}{cc} G(a) & :=\frac{1}{\sqrt{2\pi }}\int_{-\infty }^{a}e^{-\frac{x^{2}}{2}}dx, \end{array}$$

*which is the cumulative distribution function of the standard Gaussian distribution, and for any pair of distributions $\left(Q_{1},Q_{2}\right)\in P{\left(X\right)}^{2}$,*

(68) $$\begin{array}{c} V\left(Q_{1},Q_{2},\gamma \right):=\gamma {\mathrm{Var}}_{Q_{1}}\left[\iota_{1}\left(X|Q_{1},Q_{2},\gamma \right)\right]+{\mathrm{Var}}_{Q_{2}}\left[\iota_{2}\left(X|Q_{1},Q_{2},\gamma \right)\right], \end{array}$$

*where ${\mathrm{Var}}_{Q}\left[\cdot \right]$ represents the variances with respect to Q∈P(X).*

**Proof.**  The proof is provided in Appendix E. □

**Remark** **4.** *If U is a singleton (u=1), Theorem 3 reduces to $\hat{r}\left(\epsilon ,\lambda \right)=\sqrt{V(P_{1},P_{2}^{j},\gamma )}G^{-1}\left(\epsilon \right)$ for $\lambda =\hat{\lambda }\left(\epsilon \right)=\mathrm{GJS}\left(P_{1},P_{2}^{j},\gamma \right)$, which is the same result given by Zhou et al. [5].*

**Remark** **5.** *We can summarize the two terms on the right-hand side of (65) into the following single term called the canonical equation [6]:*

(69) $$\begin{array}{c} \sum_{j\in U}w\left(j\right)\lim_{n\to \infty }\Phi_{j}\left(\sqrt{n}\left(\lambda -\mathrm{GJS}\left(P_{1},P_{2}^{j},\gamma \right)\right)+r\right). \end{array}$$

*We focus on the case $\lambda =\hat{\lambda }\left(\epsilon \right)$. From Theorem 2, it holds that*

(70) $$\begin{array}{c} \sum_{\left\{j\in U:\;\mathrm{MGJS}\left(P_{1},P_{2}^{j},\gamma \right)<\lambda \right\}}w\left(j\right)\leq \epsilon \end{array}$$

*and*

(71) $$\begin{array}{c} \sum_{\left\{j\in U:\;\mathrm{MGJS}\left(P_{1},P_{2}^{j},\gamma \right)\leq \lambda \right\}}w\left(j\right)\geq \epsilon . \end{array}$$

*Here, let us consider the following canonical equation for r*

(72) $$\begin{array}{c} \sum_{j\in U}w\left(j\right)\lim_{n\to \infty }\Phi_{j}\left(\sqrt{n}\left(\hat{\lambda }\left(\epsilon \right)-\mathrm{GJS}\left(P_{1},P_{2}^{j},\gamma \right)\right)+r\right)=\epsilon . \end{array}$$

*Thus, in view of (70) and (71), this equation always has the solution r=r(ϵ). If*

(73) $$\begin{array}{c} \sum_{\left\{j\in U:\;\mathrm{GJS}\left(P_{1},P_{2}^{j},\gamma \right)=\hat{\lambda }\left(\epsilon \right)\right\}}w\left(j\right)=0, \end{array}$$

*the solution is not unique (r(ϵ)=+∞). By using the solution r(ϵ), Equation (65) with $\lambda =\hat{\lambda }\left(\epsilon \right)$ can be expressed in a simpler form*

(74) $$\begin{array}{c} \hat{r}\left(\epsilon ,\lambda \right)=r\left(\epsilon \right). \end{array}$$

## 4. Generalization to Mixed Memoryless Sources with General Mixture

In this section, we consider the classification problem in the case where $P_{1}$ does not have subclasses and $P_{2}$ is given by a general mixture model. The general mixture model considered in this problem represents an extension of the source with multiple subclasses defined in Section 2.2. Since the decision rule that achieves the maximum error exponent can be operated using only one of the training sequences, we assume in this section that only the training sequence $t_{1}$ is available. Then, we provide a characterization of the maximum error exponents in a single-letter form under this setting. First, we define the source referred to as a mixed memoryless source with general mixture [6,9] as follows. Let Θ be an arbitrary probability space with a general probability measure w(θ),θ∈Θ. Then, the probability of $x\in X^{n}$ is given by

(75) $$\begin{array}{c} P_{2}\left(x\right)=\int_{\theta \in \Theta }P_{2}^{\theta }\left(x\right)dw\left(\theta \right), \end{array}$$

where $P_{2}^{\theta }$ is a stationary and memoryless source for each θ∈Θ. That is, for $x=\left(x_{1},x_{2},\cdots ,x_{n}\right)\in X^{n}$

(76) $$\begin{array}{c} P_{2}^{\theta }\left(x\right)=\prod_{i=1}^{n}P_{2}^{\theta }\left(x_{i}\right). \end{array}$$

When a test sequence is output from $P_{2}$, the probability distribution of the sequence takes the form of (75). Here, type-I and type-II error probabilities of a test $\phi^{n}=\left(\phi_{1}^{n},\phi_{2}^{n}\right)$ are given by

(77) $$\begin{array}{c} \beta_{1}\left(\phi^{n}|P_{1},P_{2}\right)=\sum_{\left(x,t_{1}\right)\in \phi_{2}^{n}}P_{1}\left(x\right)P_{1}\left(t_{1}\right), \end{array}$$

(78) $$\begin{array}{c} \beta_{2}\left(\phi^{n}|P_{1},P_{2}\right)=\sum_{\left(x,t_{1}\right)\in \phi_{1}^{n}}P_{2}\left(x\right)P_{1}\left(t_{1}\right). \end{array}$$

**Theorem** **4.** *(First-and second-order maximum error exponents) For any pair of distributions $\left(P_{1},P_{2}\right)\in P\left(X\right)\times P_{\Theta }\left(X\right)$ and ϵ∈[0,1), we have*

(79) $$\begin{array}{c} \hat{\lambda }\left(\epsilon \right)=\sup\left\{\bar{\lambda }\;|\int_{\left\{\theta \in \Theta :\;\mathrm{GJS}\left(P_{1},P_{2}^{\theta },\gamma \right)<\bar{\lambda }\right\}}dw\left(\theta \right)\leq \epsilon \right\} \end{array}$$

*and*

(80) $$\begin{array}{cc} \hat{r}\left(\epsilon ,\lambda \right) & =\sup\left\{r\;|\int_{\left\{\theta \in \Theta :\;\mathrm{GJS}(P_{1},P_{2}^{\theta },\gamma )<\lambda \right\}}dw\left(\theta \right)+\int_{\left\{\theta \in \Theta :\;\mathrm{GJS}(P_{1},P_{2}^{\theta },\gamma )=\lambda \right\}}\Phi_{\theta }\left(r\right)dw\left(\theta \right)\leq \epsilon \right\}. \end{array}$$

**Proof.**  We can prove this theorem in the same way as Theorems 2 and 3. □

## 5. Numerical Calculation

### 5.1. First-Order Maximum Error Exponent

In this section, we present a numerical example to illustrate the first-order maximum type-I error exponent $\hat{\lambda }\left(\epsilon \right)$ characterized in Theorem 2.

**Figure 2:**
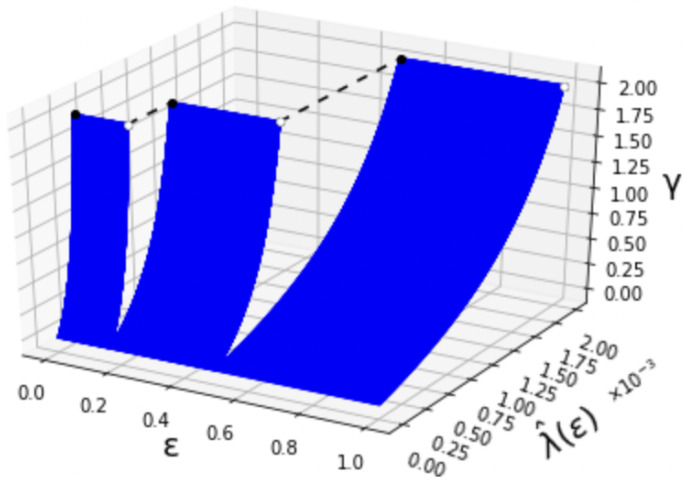
The first-order maximum type-I error exponent $\hat{\lambda }\left(\epsilon \right)$ (0<γ≤2).

**Figure 3:**
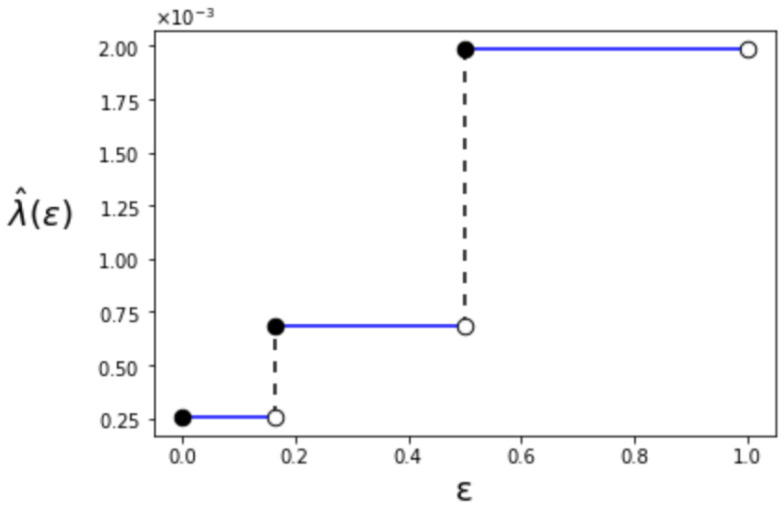
The first-order maximum type-I error exponent $\hat{\lambda }\left(\epsilon \right)$ (γ=2).

A numerical example of the first-order maximum error exponent is given by calculating the right-hand side of (64) for the following settings. We assume that X={0,1}. We fix the set of probabilities and weights

(81) $$\begin{array}{c} P_{1}=\left\{\left\{\mathrm{Bern}\left(0.389\right),v\left(1\right)=\frac{1}{3}\right\},\left\{\mathrm{Bern}\left(0.322\right),v\left(2\right)=\frac{1}{3}\right\},\left\{\mathrm{Bern}\left(0.256\right),v\left(3\right)=\frac{1}{3}\right\}\right\} \end{array}$$

and

(82) $$\begin{array}{c} P_{2}=\left\{\left\{\mathrm{Bern}\left(0.301\right),w\left(1\right)=\frac{1}{3}\right\},\left\{\mathrm{Bern}\left(0.244\right),w\left(2\right)=\frac{1}{6}\right\},\left\{\mathrm{Bern}\left(0.223\right),w\left(3\right)=\frac{1}{2}\right\}\right\}, \end{array}$$

where Bern(·) denotes the Bernoulli distribution. The relation among $\hat{\lambda }\left(\epsilon \right)$, ϵ and γ is shown in Figure 2. Additionally, for γ=2, the behavior of $\hat{\lambda }\left(\epsilon \right)$ is depicted in Figure 3. When ϵ becomes larger, the value of $\hat{\lambda }\left(\epsilon \right)$ also increases like a step function. The step increases when $\epsilon =\frac{1}{6}$ and $\epsilon =\frac{1}{2}$. We can also confirm that $\hat{\lambda }\left(\epsilon \right)$ is right-continuous in ϵ. This is due to the limit that the superior of the type-II error probability is constrained in Definition 1.

### 5.2. Second-Order Maximum Error Exponent

As in the previous subsection, we present a numerical example to illustrate the second-order maximum type-I error exponent $\hat{r}\left(\epsilon ,\lambda \right)$ characterized in Theorem 3.

**Figure 4:**
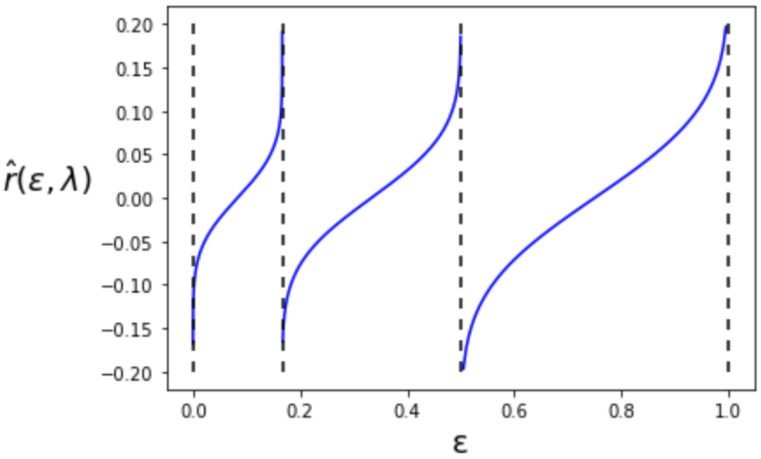
The second-order maximum type-I error exponent $\hat{r}\left(\epsilon ,\lambda \right)$.

A numerical example of the second-order maximum error exponent is given by calculating the right-hand side of (65) for the following settings. We assume that γ=2, X={0,1}. We fix $\lambda =\hat{\lambda }\left(\epsilon \right)$ and the set of probabilities and weights

(83) $$\begin{array}{c} P_{1}=\mathrm{Bern}\left(0.268\right) \end{array}$$

and

(84) $$\begin{array}{c} P_{2}=\left\{\left\{\mathrm{Bern}\left(0.301\right),w\left(1\right)=\frac{1}{3}\right\},\left\{\mathrm{Bern}\left(0.244\right),w\left(2\right)=\frac{1}{6}\right\},\left\{\mathrm{Bern}\left(0.223\right),w\left(3\right)=\frac{1}{2}\right\}\right\}, \end{array}$$

where $P_{2}$ is the same as the setting in the previous subsection. The behavior of $\hat{r}\left(\epsilon ,\lambda \right)$ is shown in Figure 4. The value of $\hat{r}\left(\epsilon ,\lambda \right)$ takes the inverse of the cumulative distribution function of the standard Gaussian for each interval of ϵ such that $0\leq \epsilon <\frac{1}{6}$, $\frac{1}{6}\leq \epsilon <\frac{1}{2}$ and $\frac{1}{2}\leq \epsilon <1$. In contrast to the first-order $\hat{\lambda }\left(\epsilon \right)$, $\hat{r}\left(\epsilon ,\lambda \right)$ is no longer right continuous in ϵ.

## 6. Conclusions

For binary classification of sources with multiple subclasses, we characterized the first- and second-order maximum error exponents. First, we revealed the first-order maximum error exponent in the case where $P_{1}$ and $P_{2}$ are sources with multiple subclasses. In order to derive this representation, we gave a classifier which achieves the asymptotically maximum error exponent in the class of deterministic classifiers for sources with multiple subclasses.

Next, we showed the second-order maximum error exponent in the case where only one of sources has subclasses. The most important key technique to derive the second-order maximum error exponent is to apply the Berry–Esseen theorem [8] instead of the weak law of large numbers. One may wonder whether we can also derive the second-order approximation in the case where $P_{1}$ is also a source with multiple subclasses. To this end, we need to evaluate Lemma 2 more rigorously. This is future work.

In addition, for binary classification using only a training sequence generated from $P_{1}$ in the case where $P_{1}$ does not have subclasses and $P_{2}$ is given by a general mixture model, we generalized the analysis for the first- and second-order error exponents. From these results, we revealed the asymptotic performance limits of statistical classification for sources with multiple subclasses.

In this paper, we considered a binary classification problem, but in practice, multiclass classification is of importance. In the case where each class is a memoryless source (without multiple subclasses), the first- and second-order maximum error exponents were analyzed in [5]. Extending the obtained results to multiclass classification for sources with multiple subclasses is also a subject of future studies.

## Data Availability

Not applicable.

## Appendix A

**Proof** (**Proof** **of** **Equation (33)**). We shall show

(A1) $$\begin{array}{c} ND(q_{t_{1}}\left|\right|{\tilde{P}}_{1})+nD(q_{x}\left|\right|{\tilde{P}}_{1})=ND(q_{t_{1}}\left|\right|q_{y_{1}})+nD(q_{x}\left|\right|q_{y_{1}})+n\left(1+\gamma \right)D\left(\frac{\gamma q_{t_{1}}+q_{x}}{1+\gamma }\left|\right|{\tilde{P}}_{1}\right). \end{array}$$

The left-hand side of (A1) is expanded as follows:

(A2) $$\begin{array}{cc} \; & ND(q_{t_{1}}\left|\right|{\tilde{P}}_{1})+nD(q_{x}\left|\right|{\tilde{P}}_{1})\\ \; & =n\gamma \sum_{x\in X}q_{t_{1}}\left(x\right)\log\frac{q_{t_{1}}\left(x\right)}{{\tilde{P}}_{1}\left(x\right)}+n\sum_{x\in X}q_{x}\left(x\right)\log\frac{q_{x}\left(x\right)}{{\tilde{P}}_{1}\left(x\right)}-n\gamma \sum_{x\in X}q_{t_{1}}\left(x\right)\log\frac{\gamma q_{t_{1}}\left(x\right)+q_{x_{1}}\left(x\right)}{1+\gamma }\\ \; & \;\;\;\;\;\;\;\;\;\;\;\;+n\gamma \sum_{x\in X}q_{t_{1}}\left(x\right)\log\frac{\gamma q_{t_{1}}\left(x\right)+q_{x}\left(x\right)}{1+\gamma }-n\sum_{x\in X}q_{x}\left(x\right)\log\frac{\gamma q_{t_{1}}\left(x\right)+q_{x}\left(x\right)}{1+\gamma }\\ \; & \;\;\;\;\;\;\;\;\;\;\;\;+n\sum_{x\in X}q_{x}\left(x\right)\log\frac{\gamma q_{t_{1}}\left(x\right)+q_{x_{1}}\left(x\right)}{1+\gamma }\\ \; & =n\gamma E_{q_{t_{1}}}\left[\log\frac{\left(1+\gamma \right)q_{t_{1}}\left(x\right)}{\gamma q_{t_{1}}\left(x\right)+q_{x}\left(x\right)}\right]+nE_{q_{x}}\left[\log\frac{\left(1+\gamma \right)q_{x}\left(x\right)}{\gamma q_{t_{1}}\left(x\right)+q_{x}\left(x\right)}\right]\\ \; & \;\;\;\;\;\;\;\;\;\;\;\;-n\sum_{x\in X}\left(\gamma q_{t_{1}}\left(x\right)+q_{x}\left(x\right)\right)\log{\tilde{P}}_{1}\left(x\right)+n\sum_{x\in X}\left(\gamma q_{t_{1}}\left(x\right)+q_{x}\left(x\right)\right)\log\frac{\gamma q_{t_{1}}\left(x\right)+q_{x}\left(x\right)}{1+\gamma }\\ \; & =ND(q_{t_{1}}\left|\right|q_{y_{1}})+nD(q_{x}\left|\right|q_{y_{1}})+n\left(1+\gamma \right)D\left(\frac{\gamma q_{t_{1}}+q_{x}}{1+\gamma }\left|\right|{\tilde{P}}_{1}\right). \end{array}$$

Therefore, we obtain Equation (33). □

## Appendix B

**Proof** **of** **Lemma 2)**. Applying the Taylor expansion to $\mathrm{GJS}(q_{t_{1}^{i}},q_{x},\gamma )$ around $\left(Q_{1}^{i},Q_{2}\right)$ for any $q_{x}\in B_{n}\left(Q_{2}\right),q_{t_{1}^{i}}\in B_{N}\left(Q_{1}^{i}\right)\;\left(\forall i\in S\right)$, we obtain

(A3) $$\begin{array}{c} \mathrm{GJS}\left(q_{t_{1}^{i}},q_{x},\gamma \right)=\mathrm{GJS}\left(Q_{1}^{i},Q_{2},\gamma \right)+O\left(\sqrt{\frac{\log n}{n}}\right). \end{array}$$

Therefore by (14) for any $q_{x}\in B_{n}\left(Q_{2}\right)$, $q_{t_{1}^{i}}\in B_{N}\left(Q_{1}^{i}\right)\;\left(\forall i\in S\right)$,

(A4) $$\begin{array}{cc} \; & \gamma D\left(q_{t_{1}^{i}}\left|\right|\frac{q_{x}+\gamma q_{t_{1}^{i}}}{1+\gamma }\right)+D\left(q_{x}\left|\right|\frac{q_{x}+\gamma q_{t_{1}^{i}}}{1+\gamma }\right)\\ & \;\;\;\;\;\;\;\;\to \gamma D\left(Q_{1}^{i}\left|\right|\frac{Q_{2}+\gamma Q_{1}^{i}}{1+\gamma }\right)+D\left(Q_{2}\left|\right|\frac{Q_{2}+\gamma Q_{1}^{i}}{1+\gamma }\right)\;\;\;\;\left(n\to \infty \right) \end{array}$$

holds, and the convergence is uniform in i∈S. Since for any $\left(Q_{1},Q_{2}\right)\in P_{S}\left(X\right)\times P(X)$, $i^{*}\left(Q_{1},Q_{2}\right)$ was given in the form

(A5) $$\begin{array}{c} i^{*}\left(Q_{1},Q_{2}\right)=\underset{i\in S}{arg min}\gamma D\left(Q_{1}\left|\right|\frac{Q_{2}+\gamma Q_{1}}{1+\gamma }\right)+D\left(Q_{2}\left|\right|\frac{Q_{2}+\gamma Q_{1}}{1+\gamma }\right), \end{array}$$

we obtain

(A6) $$\begin{array}{c} i^{*}\left(q_{t_{1}},q_{x}\right)\to i^{*}\left(Q_{1},Q_{2}\right)\;\;\;\;\left(n\to \infty \right). \end{array}$$

□

## Appendix C

**Proof** **of** **Lemma 5**. It follows from (56) that

$$\begin{array}{cc} \; & \exp\left\{-n\lambda \right\} \end{array}$$

(A7) $$\begin{array}{c} \geq \sum_{\left(x,t_{1},t_{2}\right)\in \Omega_{2}^{n}}{\tilde{P}}_{1}\left(x\right)\prod_{r=1}^{s}{\tilde{P}}_{1}^{r}\left(t_{1}^{r}\right)\prod_{j=1}^{u}{\tilde{P}}_{2}^{j}\left(t_{2}^{j}\right)\\ =\sum_{\left(x,t_{1},t_{2}\right)\in \Omega_{2}^{n}}\sum_{i=1}^{s}{\tilde{P}}_{1}^{i}\left(x\right)\prod_{r=1}^{s}{\tilde{P}}_{1}^{r}\left(t_{1}^{r}\right)\prod_{j=1}^{u}{\tilde{P}}_{2}^{j}\left(t_{2}^{j}\right)v\left(i\right)\\ \geq \sum_{\left(x,t_{1},t_{2}\right)\in \Omega_{2}^{n}}{\tilde{P}}_{1}^{i}\left(x\right)\prod_{r=1}^{s}{\tilde{P}}_{1}^{r}\left(t_{1}^{r}\right)\prod_{j=1}^{u}{\tilde{P}}_{2}^{j}\left(t_{2}^{j}\right)v\left(i\right)\;\;\;\;\left(\forall i\in S,v\left(i\right)>0\right)\\ =\sum_{\left(q_{x},q_{t_{1}},q_{t_{2}}\right)\in \Gamma \left(\Omega_{2}^{n}\right)}{\tilde{P}}_{1}^{i}\left(T_{n}\left(q_{x}\right)\right)\prod_{r=1}^{s}{\tilde{P}}_{1}^{r}\left(T_{N}\left(q_{t_{1}^{r}}\right)\right)\prod_{j=1}^{u}{\tilde{P}}_{2}^{j}\left(T_{N}\left(q_{t_{2}^{j}}\right)\right)v\left(i\right)\\ \geq {\tilde{P}}_{1}^{i}\left(T_{n}\left(q_{x}\right)\right)\prod_{r=1}^{s}{\tilde{P}}_{1}^{r}\left(T_{N}\left(q_{t_{1}^{r}}\right)\right)\prod_{j=1}^{u}{\tilde{P}}_{2}^{j}\left(T_{N}\left(q_{t_{2}^{j}}\right)\right)v\left(i\right)\;\;\;\;\;\left(\forall \left(q_{x},q_{t_{1}},q_{t_{2}}\right)\in \Gamma \left(\Omega_{2}^{n}\right)\right). \end{array}$$

Using (30) on the right-hand side of (A7), we obtain

(A8) $$\begin{array}{cc} \; & \exp\left\{-n\lambda \right\}\\ \; & \geq \exp\left\{-n\rho (n)\right\}\exp\left\{-n\left[D(q_{x}\left|\right|{\tilde{P}}_{1}^{i})+\sum_{r=1}^{s}\gamma D(q_{t_{1}^{r}}\left|\right|{\tilde{P}}_{1}^{r})+\sum_{j=1}^{u}\gamma D(q_{t_{2}^{j}}\left|\right|{\tilde{P}}_{2}^{j})\right]\right\}, \end{array}$$

where $\rho \left(n\right)=\frac{|X|\log\left(n+1\right)+\left(s+u\right)|X|\log\left(N+1\right)-\log v^{*}}{n}$. Taking the negative logarithm of both sides and divide by *n* in (A8), we have

(A9) $$\begin{array}{cc} \; & \lambda -\rho \left(n\right)\leq D(q_{x}\left|\right|{\tilde{P}}_{1}^{i})+\sum_{r=1}^{s}\gamma D(q_{t_{1}^{r}}\left|\right|{\tilde{P}}_{1}^{r})+\sum_{j=1}^{u}\gamma D(q_{t_{2}^{j}}\left|\right|{\tilde{P}}_{2}^{j}). \end{array}$$

Since $\left({\tilde{P}}_{1}^{i},{\tilde{P}}_{1}^{r},{\tilde{P}}_{2}^{j}\right)\in P{\left(X\right)}^{3}$ is arbitrary in (A9), we can set the *i*-th subclass as ${\tilde{P}}_{1}^{i}=q_{xt_{1}^{i}}=q_{y_{1}^{i}}$ and the other as ${\tilde{P}}_{1}^{r}=q_{t_{1}^{r}}$, r≠i. Furthermore, we set ${\tilde{P}}_{2}^{j}=q_{t_{2}^{j}},\forall j\in U$. Then we obtain

(A10) $$\begin{array}{cc} \lambda -\rho (n) & \leq D(q_{x}\left|\right|q_{y_{1}^{i}})+\gamma D(q_{t_{1}^{i}}\left|\right|q_{y_{1}^{i}})\;\;\;\;\left(\forall \left(q_{x},q_{t_{1}},q_{t_{2}}\right)\in \Gamma \left(\Omega_{2}^{n}\right),\forall i\in S\right)\\ ⟺\lambda -\rho (n) & \leq \min_{i\in S}\left\{D(q_{x}\left|\right|q_{y_{1}^{i}})+\gamma D(q_{t_{1}^{i}}\left|\right|q_{y_{1}^{i}})\right\}\;\;\;\;\left(\forall \left(q_{x},q_{t_{1}},q_{t_{2}}\right)\in \Gamma \left(\Omega_{2}^{n}\right)\right). \end{array}$$

Therefore by (18), (A10) implies that $\Omega_{2}^{n}$ is constrained in

(A11) $$\begin{array}{c} {\bar{\Lambda }}_{2}^{n}:=\left\{\left(x,t_{1},t_{2}\right)\;|\;\mathrm{MGJS}\left(q_{t_{1}},q_{x},\gamma \right)\geq \lambda -\rho \left(n\right)\right\}, \end{array}$$

and for any pair of distributions $P=\left(P_{1},P_{2}\right)\in P_{S}\left(X\right)\times P_{U}\left(X\right)$, we obtain

(A12) $$\begin{array}{c} \Omega_{2}^{n}\subset {\bar{\Lambda }}_{2}^{n}\;\;\Rightarrow \;\;{\bar{\Lambda }}_{1}^{n}\subset \Omega_{1}^{n}\;\;\Rightarrow \;\;\beta_{2}\left({\bar{\Lambda }}^{n}|P\right)\leq \beta_{2}\left(\Omega^{n}|P\right). \end{array}$$

□

## Appendix D

**Proof** **of** **Corollary 1**. It follows from (58) that

(A13) $$\begin{array}{cc} \exp(-n\lambda ) & \geq \beta_{1}\left(\phi^{n}|{\tilde{P}}_{1},{\tilde{P}}_{2}\right)\\ \; & \geq \frac{1}{n}\beta_{1}\left(\Omega^{n}|{\tilde{P}}_{1},{\tilde{P}}_{2}\right), \end{array}$$

where Lemma 4 with κ=1/n guarantees the existence of such a type-based test $\Omega^{n}$ satisfying (A13) for $\left({\tilde{P}}_{1},{\tilde{P}}_{2}\right)\in P_{S}\left(X\right)\times P_{U}\left(X\right)$ and (55) for $\left(P_{1},P_{2}\right)\in P_{S}\left(X\right)\times P_{U}\left(X\right)$ (cf. Remark 3). Then, the type-I error probability of $\Omega^{n}$ satisfies

(A14) $$\begin{array}{cc} \beta_{1}\left(\Omega^{n}|{\tilde{P}}_{1},{\tilde{P}}_{2}\right) & \leq n\exp(-n\lambda )\\ \; & =\exp\left(-n\left\{\lambda -\frac{\log n}{n}\right\}\right)\\ \; & =\exp(-n\lambda^{\prime }), \end{array}$$

where $\lambda^{\prime }=\lambda -\frac{\log n}{n}$. From Lemma 5, the type-II error probability of $\Omega^{n}$ satisfies

(A15) $$\begin{array}{c} \beta_{2}\left(\Omega^{n}|P_{1},P_{2}\right)\geq P_{2}\left\{\mathrm{MGJS}\left(q_{t_{1}},q_{x},\gamma \right)<\lambda^{\prime }-\rho \left(n\right)\right\}. \end{array}$$

Combining (A15) and (55) for $\left(P_{1},P_{2}\right)\in P_{S}\left(X\right)\times P_{U}\left(X\right)$, we have

(A16) $$\begin{array}{cc} \beta_{2}\left(\phi^{n}|P_{1},P_{2}\right) & \geq \left(1-\frac{1}{n}\right)P_{2}\left\{\mathrm{MGJS}\left(q_{t_{1}},q_{x},\gamma \right)<\lambda^{\prime }-\rho \left(n\right)\right\}\\ \; & =\left(1-\frac{1}{n}\right)P_{2}\left\{\mathrm{MGJS}\left(q_{t_{1}},q_{x},\gamma \right)+\rho \left(n\right)+\frac{\log n}{n}<\lambda \right\}, \end{array}$$

establishing (59). □

## Appendix E

**Proof** **of** **Theorem 3**. We divide the proof of Theorem 3 into two parts: the achievability (direct) part and the converse part. First, for preliminaries, we define the following quantity used in the proof: For $\left(Q_{1},Q_{2}\right)\in P{\left(X\right)}^{2}$,

(A17) $$\begin{array}{cc} T(Q_{1},Q_{2},\gamma ) & :=\gamma E_{Q_{1}}\left[|\iota_{1}\left(X|Q_{1},Q_{2},\gamma \right)-E_{Q_{1}}\left[\iota_{1}\left(X|Q_{1},Q_{2},\gamma \right)\right]{|}^{3}\right]\\ & \;\;\;\;\;\;\;\;\;\;\;\;\;\;\;+E_{Q_{2}}\left[|\iota_{2}\left(X|Q_{1},Q_{2},\gamma \right)-E_{Q_{2}}\left[\iota_{2}\left(X|Q_{1},Q_{2},\gamma \right)\right]{|}^{3}\right]. \end{array}$$

Next, we give a lemma that is important in the proof of Theorem 3. □

**Lemma** **A1** (The Berry–Esseen theorem [8]). *Let $X_{k},k=1,...,n$ be independent with*

(A18) $$\begin{array}{cc} \mu_{k} & =E[X_{k}], \end{array}$$

(A19) $$\begin{array}{cc} \sigma^{2} & =\sum_{k=1}^{n}\mathrm{Var}\left[X_{k}\right], \end{array}$$

(A20) $$\begin{array}{cc} T & =\sum_{k=1}^{n}E[|X_{k}-\mu_{k}{|}^{3}], \end{array}$$

(A21) $$\begin{array}{cc} Q(x) & =\int_{x}^{\infty }\frac{1}{\sqrt{2\pi }}\exp\left(-\frac{t^{2}}{2}\right)dt. \end{array}$$

*Then for any −∞<λ<∞, it holds that*

(A22)
$$\begin{array}{c} \left|P\left[\sum_{k=1}^{n}\left(X_{k}-\mu_{k}\right)\geq \lambda \sigma \right]-Q\left(\lambda \right)\right|\leq \frac{6T}{\sigma^{3}}. \end{array}$$

### Appendix E.1. Achievability Part

In the achievability proof, we use the following test $\Lambda^{n}=\left(\Lambda_{1}^{n},\Lambda_{2}^{n}\right)$:

(A23) $$\begin{array}{c} \Lambda_{2}^{n}=\left\{\left(x,t_{1},t_{2}\right)\;|\;\mathrm{GJS}\left(q_{t_{1}},q_{x},\gamma \right)\geq \tilde{\lambda }+\frac{r}{\sqrt{n}}\right\}, \end{array}$$

where $\mathrm{MGJS}(q_{t_{1}},q_{x},\gamma )$ in (17) is now $\mathrm{GJS}(q_{t_{1}},q_{x},\gamma )$ because the first source is assumed to be a stationary and memoryless source. Fix any

(A24) $$\begin{array}{c} r<\sup\left\{r\;|\sum_{\left\{j\in U:\;\mathrm{GJS}(P_{1},P_{2}^{j},\gamma )<\lambda \right\}}w\left(j\right)+\sum_{\left\{j\in U:\;\mathrm{GJS}(P_{1},P_{2}^{j},\gamma )=\lambda \right\}}\Phi_{j}\left(r\right)w\left(j\right)\leq \epsilon \right\}. \end{array}$$

Then, for any pair of distributions $\left(P_{1},P_{2}\right)\in P\left(X\right)\times P_{U}\left(X\right)$ and for all pairs of distributions $\left({\tilde{P}}_{1},{\tilde{P}}_{2}\right)\in P\left(X\right)\times P_{U}\left(X\right)$, we show

(A25) $$\begin{array}{cc} \beta_{1}\left(\Lambda^{n}|{\tilde{P}}_{1},{\tilde{P}}_{2}\right) & \leq \exp(-n\lambda -\sqrt{n}r), \end{array}$$

(A26) $$\begin{array}{cc} \underset{n\to \infty }{lim sup}\beta_{2}\left(\Lambda^{n}|P_{1},P_{2}\right) & \leq \epsilon . \end{array}$$

The test $\Lambda^{n}$ defined by (A23) is the same as the test defined by (17) with replacing $\tilde{\lambda }$ with $\tilde{\lambda }+\frac{r}{\sqrt{n}}$ and *s* with 1, and so (A25) can be derived from the argument in Section 3.3.1. Therefore, we will prove only (A26). We use the set $B_{n}\left(Q\right),Q\in P\left(X\right)$ defined by (37). An upper bound on the type-II error probability of the test $\Lambda^{n}$, defined by (A23), for any pair of distributions $\left(P_{1},P_{2}\right)\in P\left(X\right)\times P_{U}\left(X\right)$ can be evaluated as follows:

(A27) $$\begin{array}{cc} \; & \underset{n\to \infty }{lim sup}\beta_{2}\left(\Lambda^{n}|P_{1},P_{2}\right)\\ \; & =\underset{n\to \infty }{lim sup}P_{2}\left\{\mathrm{GJS}\left(q_{t_{1}},q_{x},\gamma \right)\leq \lambda +\frac{r}{\sqrt{n}}+\eta \left(n\right)\right\}\\ \; & =\underset{n\to \infty }{lim sup}\sum_{j=1}^{u}w\left(j\right)P_{2}^{j}\left\{\mathrm{GJS}\left(q_{t_{1}},q_{x},\gamma \right)\leq \lambda +\frac{r}{\sqrt{n}}+\eta \left(n\right)\right\}\\ \; & \leq \underset{n\to \infty }{lim sup}\sum_{j=1}^{u}w\left(j\right)P_{2}^{j}\left\{\mathrm{GJS}\left(q_{t_{1}},q_{x},\gamma \right)\leq \lambda +\frac{r}{\sqrt{n}}+\eta \left(n\right),t_{1}\in B_{N}\left(P_{1}\right),x\in B_{n}\left(P_{2}^{i}\right)\right\}\\ \; & \;\;\;\;\;\;\;\;\;\;\;\;\;\;\;+\underset{n\to \infty }{lim sup}\sum_{j=1}^{u}w\left(j\right)P_{2}^{j}\left\{t_{1}\notin B_{N}\left(P_{1}\right)\;\mathrm{or}\;x\notin B_{n}\left(P_{2}^{i}\right)\right\}\\ \; & =\underset{n\to \infty }{lim sup}\sum_{j=1}^{u}w\left(j\right)P_{2}^{j}\{\frac{1}{n}\sum_{i=1}^{N}\iota_{1}\left(t_{1,i}|P_{1},P_{2}^{j},\gamma \right)+\frac{1}{n}\sum_{i=1}^{n}\iota_{2}\left(x_{i}|P_{1},P_{2}^{j},\gamma \right)\leq \lambda +\frac{r}{\sqrt{n}} \end{array}$$

(A28) $$\begin{array}{cc} \; & \;\;\;\;\;\;\;\;\;\;\;\;\;\;\;+O\left(\frac{\log n}{n}\right),t_{1}\in B_{N}\left(P_{1}\right),x\in B_{n}\left(P_{2}^{i}\right)\}\\ \; & \leq \underset{n\to \infty }{lim sup}\sum_{j=1}^{u}w\left(j\right)P_{2}^{j}\{\frac{1}{n}\sum_{i=1}^{N}\iota_{1}\left(t_{1,i}|P_{1},P_{2}^{j},\gamma \right)+\frac{1}{n}\sum_{i=1}^{n}\iota_{2}\left(x_{i}|P_{1},P_{2}^{j},\gamma \right)\leq \lambda +\frac{r}{\sqrt{n}}\\ \; & \;\;\;\;\;\;\;\;\;\;\;\;\;\;\;+O\left(\frac{\log n}{n}\right)\}\\ \; & =\underset{n\to \infty }{lim sup}\sum_{j=1}^{u}w\left(j\right)P_{2}^{j}\{\sum_{i=1}^{N}\left\{\iota_{1}\left(t_{1,i}|P_{1},P_{2}^{j},\gamma \right)-E_{P_{1}}\left[\iota_{1}\left(X|P_{1},P_{2}^{j},\gamma \right)\right]\right\}\\ \; & \;\;\;\;\;\;\;\;\;\;\;\;\;\;\;+\sum_{i=1}^{n}\left\{\iota_{2}\left(x_{i}|P_{1},P_{2}^{j},\gamma \right)-E_{P_{2}}\left[\iota_{2}\left(X|P_{1},P_{2}^{j},\gamma \right)\right]\right\}\leq n(\lambda +\frac{r}{\sqrt{n}}-\mathrm{GJS}\left(P_{1},P_{2}^{j},\gamma \right)\\ \; & \;\;\;\;\;\;\;\;\;\;\;\;\;\;\;+O\left(\frac{\log n}{n}\right))\} \end{array}$$

where (A27) follows from Lemma 3 and the proof of (A28) is provided in Appendix F.

(A29) $$\begin{array}{cc} \; & \underset{n\to \infty }{lim sup}\beta_{2}\left(\Lambda^{n}|P_{1},P_{2}\right)\\ \; & \leq \underset{n\to \infty }{lim sup}\sum_{j=1}^{u}w\left(j\right)\{G\left(\left[\lambda +\frac{r}{\sqrt{n}}-\mathrm{GJS}\left(P_{1},P_{2}^{j},\gamma \right)+O\left(\frac{\log n}{n}\right)\right]\sqrt{\frac{n}{V(P_{1},P_{2}^{j},\gamma )}}\right) \end{array}$$

(A30) $$\begin{array}{cc} \; & \;\;\;\;\;\;\;\;\;\;\;\;\;\;\;+\frac{6T(P_{1},P_{2}^{j},\gamma )}{\sqrt{n{\left(V\left(P_{1},P_{2}^{j},\gamma \right)\right)}^{3}}}\}\\ \; & =\underset{n\to \infty }{lim sup}\sum_{j=1}^{u}w\left(j\right)G\left(\left[\lambda +\frac{r}{\sqrt{n}}-\mathrm{GJS}\left(P_{1},P_{2}^{j},\gamma \right)+O\left(\frac{\log n}{n}\right)\right]\sqrt{\frac{n}{V(P_{1},P_{2}^{j},\gamma )}}\right)\\ \; & \leq \sum_{j=1}^{u}w\left(j\right)\underset{n\to \infty }{lim sup}G\left(\left[\lambda +\frac{r}{\sqrt{n}}-\mathrm{GJS}\left(P_{1},P_{2}^{j},\gamma \right)+O\left(\frac{\log n}{n}\right)\right]\sqrt{\frac{n}{V(P_{1},P_{2}^{j},\gamma )}}\right), \end{array}$$

where (A29) and the last inequality are derived from Lemma A1 and Fatou’s lemma, respectively. Here,

(A31) $$\begin{array}{cc} \; & \underset{n\to \infty }{lim sup}G\left(\left[\lambda +\frac{r}{\sqrt{n}}-\mathrm{GJS}\left(P_{1},P_{2}^{j},\gamma \right)+O\left(\frac{\log n}{n}\right)\right]\sqrt{\frac{n}{V(P_{1},P_{2}^{j},\gamma )}}\right)\\ & =\left\{\begin{array}{c} \;\;\;\;\;\;\;\;\;\;\;\;\;\;1\;\;\;\;\;\;\;\;\;\;\;\;\;\;\;\;\;\;\mathrm{for}\;j\in U:\;\mathrm{GJS}(P_{1},P_{2}^{j},\gamma )<\lambda \\ G\left(\frac{r}{\sqrt{V(P_{1},P_{2}^{j},\gamma )}}\right)\;\;\;\;\;\;\;\mathrm{for}\;j\in U:\;\mathrm{GJS}\left(P_{1},P_{2}^{j},\gamma \right)=\lambda \\ \;\;\;\;\;\;\;\;\;\;\;\;\;\;0\;\;\;\;\;\;\;\;\;\;\;\;\;\;\;\;\;\;\mathrm{for}\;j\in U:\;\mathrm{GJS}(P_{1},P_{2}^{j},\gamma )>\lambda \end{array}\right.. \end{array}$$

Therefore,

(A32) $$\begin{array}{cc} \; & \underset{n\to \infty }{lim sup}\beta_{2}\left(\Lambda^{n}|P_{1},P_{2}\right)\\ \; & \leq \sum_{\left\{j\in U:\;\mathrm{GJS}(P_{1},P_{2}^{j},\gamma )<\lambda \right\}}w\left(j\right)+\sum_{\left\{j\in U:\;\mathrm{GJS}(P_{1},P_{2}^{j},\gamma )=\lambda \right\}}G\left(\frac{r}{\sqrt{V(P_{1},P_{2}^{j},\gamma )}}\right)w\left(j\right)\\ \; & =\sum_{\left\{j\in U:\;\mathrm{GJS}(P_{1},P_{2}^{j},\gamma )<\lambda \right\}}w\left(j\right)+\sum_{\left\{j\in U:\;\mathrm{GJS}(P_{1},P_{2}^{j},\gamma )=\lambda \right\}}\Phi \left(r\right)w\left(j\right). \end{array}$$

Thus, by (A24), we can see that

(A33) $$\begin{array}{cc} \underset{n\to \infty }{lim sup}\beta_{2}\left(\Lambda^{n}|P_{1},P_{2}\right) & \leq \epsilon . \end{array}$$

### Appendix E.2. Converse Part

For any pair of distributions $\left(P_{1},P_{2}\right)\in P\left(X\right)\times P_{U}\left(X\right)$ and for all pairs of distributions $\left({\tilde{P}}_{1},{\tilde{P}}_{2}\right)\in P\left(X\right)\times P_{U}\left(X\right)$, fix any test $\phi^{n}$ satisfying that

(A34) $$\begin{array}{cc} \beta_{1}\left(\phi^{n}|{\tilde{P}}_{1},{\tilde{P}}_{2}\right) & \leq \exp(-n\lambda -\sqrt{n}r), \end{array}$$

(A35) $$\begin{array}{cc} \underset{n\to \infty }{lim sup}\beta_{2}\left(\phi^{n}|P_{1},P_{2}\right) & \leq \epsilon . \end{array}$$

We show

(A36) $$\begin{array}{c} r\leq \sup\left\{r\;|\sum_{\left\{j\in U:\;\mathrm{GJS}(P_{1},P_{2}^{j},\gamma )<\lambda \right\}}w\left(j\right)+\sum_{\left\{j\in U:\;\mathrm{GJS}(P_{1},P_{2}^{j},\gamma )=\lambda \right\}}\Phi_{j}\left(r\right)w\left(j\right)\leq \epsilon \right\}. \end{array}$$

To prove (A36), we use Corollary 1 with replacing λ with $\lambda +\frac{r}{\sqrt{n}}$. A lower bound on the type-II error probability of the test $\phi^{n}$ for any pair of distributions $\left(P_{1},P_{2}\right)\in P\left(X\right)\times P_{U}\left(X\right)$ can be evaluated as follows:

$$\begin{array}{cc} \; & \underset{n\to \infty }{lim sup}\beta_{2}\left(\phi^{n}|P_{1},P_{2}\right)\\ \; & \geq \underset{n\to \infty }{lim sup}\left(1-\frac{1}{n}\right)\sum_{j=1}^{u}w\left(j\right)P_{2}^{j}\left\{\mathrm{GJS}\left(q_{t_{1}},q_{x},\gamma \right)+\rho \left(n\right)+O\left(\frac{\log n}{n}\right)<\lambda +\frac{r}{\sqrt{n}}\right\}\\ \; & \geq \underset{n\to \infty }{lim sup}\left(1-\frac{1}{n}\right)\sum_{j=1}^{u}w\left(j\right)P_{2}^{j}\{\mathrm{GJS}\left(q_{t_{1}},q_{x},\gamma \right)+\rho \left(n\right)+O\left(\frac{\log n}{n}\right)<\lambda +\frac{r}{\sqrt{n}}, \end{array}$$

(A37) $$\begin{array}{c} \;\;\;\;\;\;\;\;\;\;\;\;\;\;\;t_{1}\in B_{N}\left(P_{1}\right),x\in B_{n}\left(P_{2}^{j}\right)\}\\ =\underset{n\to \infty }{lim sup}\left(1-\frac{1}{n}\right)\sum_{j=1}^{u}w\left(j\right)P_{2}^{j}\left\{\frac{1}{n}\sum_{i=1}^{N}\iota_{1}\left(t_{1,i}|P_{1},P_{2}^{j},\gamma \right)+\frac{1}{n}\sum_{i=1}^{n}\iota_{2}\left(x_{i}|P_{1},P_{2}^{j},\gamma \right)<\lambda +\frac{r}{\sqrt{n}}\right. \end{array}$$

(A38) $$\begin{array}{c} \;\;\;\;\;\;\;\;\;\;\;\;\;\;\;\left.+O\left(\frac{\log n}{n}\right)\right\}-\underset{n\to \infty }{lim sup}P_{2}^{j}\left\{t_{1}\in B_{N}\left(P_{1}\right),x\in B_{n}\left(P_{2}^{j}\right)\right\}\\ \geq \underset{n\to \infty }{lim sup}\left(1-\frac{1}{n}\right)\sum_{j=1}^{u}w\left(j\right)\{G\left(\left[\lambda +\frac{r}{\sqrt{n}}-\mathrm{GJS}\left(P_{1},P_{2}^{j},\gamma \right)+O\left(\frac{\log n}{n}\right)\right]\sqrt{\frac{n}{V(P_{1},P_{2}^{j},\gamma )}}\right) \end{array}$$

(A39) $$\begin{array}{cc} \; & \;\;\;\;\;\;\;\;\;\;\;\;\;\;\;-\frac{6T(P_{1},P_{2}^{j},\gamma )}{\sqrt{n{\left(V\left(P_{1},P_{2}^{j},\gamma \right)\right)}^{3}}}\}\\ \; & =\underset{n\to \infty }{lim sup}\sum_{j=1}^{u}w\left(j\right)\{G\left(\left[\lambda +\frac{r}{\sqrt{n}}-\mathrm{GJS}\left(P_{1},P_{2}^{j},\gamma \right)+O\left(\frac{\log n}{n}\right)\right]\sqrt{\frac{n}{V(P_{1},P_{2}^{j},\gamma )}}\right)\\ \; & \geq \underset{n\to \infty }{lim inf}\sum_{j=1}^{u}w\left(j\right)G\left(\left[\lambda +\frac{r}{\sqrt{n}}-\mathrm{GJS}\left(P_{1},P_{2}^{j},\gamma \right)+O\left(\frac{\log n}{n}\right)\right]\sqrt{\frac{n}{V(P_{1},P_{2}^{j},\gamma )}}\right)\\ \; & \geq \sum_{j=1}^{u}w\left(j\right)\underset{n\to \infty }{lim inf}G\left(\left[\lambda +\frac{r}{\sqrt{n}}-\mathrm{GJS}\left(P_{1},P_{2}^{j},\gamma \right)+O\left(\frac{\log n}{n}\right)\right]\sqrt{\frac{n}{V(P_{1},P_{2}^{j},\gamma )}}\right), \end{array}$$

where (A37) and (38) follow from Lemma 3 and Lemma A1, respectively. The last inequality is due to Fatou’s lemma. Here,

(A40) $$\begin{array}{cc} \; & \underset{n\to \infty }{lim inf}G\left(\left[\lambda +\frac{r}{\sqrt{n}}-\mathrm{GJS}\left(P_{1},P_{2}^{j},\gamma \right)+O\left(\frac{\log n}{n}\right)\right]\sqrt{\frac{n}{V(P_{1},P_{2}^{j},\gamma )}}\right)\\ & =\left\{\begin{array}{c} \;\;\;\;\;\;\;\;\;\;\;\;\;\;1\;\;\;\;\;\;\;\;\;\;\;\;\;\;\;\;\;\;\mathrm{for}\;j\in U:\;\mathrm{GJS}(P_{1},P_{2}^{j},\gamma )<\lambda \\ G\left(\frac{r}{\sqrt{V(P_{1},P_{2}^{j},\gamma )}}\right)\;\;\;\;\;\;\;\mathrm{for}\;j\in U:\;\mathrm{GJS}\left(P_{1},P_{2}^{j},\gamma \right)=\lambda \\ \;\;\;\;\;\;\;\;\;\;\;\;\;\;0\;\;\;\;\;\;\;\;\;\;\;\;\;\;\;\;\;\;\mathrm{for}\;j\in U:\;\mathrm{GJS}(P_{1},P_{2}^{j},\gamma )>\lambda \end{array}\right.. \end{array}$$

Therefore,

(A41) $$\begin{array}{cc} \; & \underset{n\to \infty }{lim sup}\beta_{2}\left(\phi^{n}|P_{1},P_{2}\right)\\ \; & \geq \sum_{\left\{j\in U:\;\mathrm{GJS}(P_{1},P_{2}^{j},\gamma )<\lambda \right\}}w\left(j\right)+\sum_{\left\{j\in U:\;\mathrm{GJS}(P_{1},P_{2}^{j},\gamma )=\lambda \right\}}G\left(\frac{r}{\sqrt{V(P_{1},P_{2}^{j},\gamma )}}\right)w\left(j\right)\\ \; & =\sum_{\left\{j\in U:\;\mathrm{GJS}(P_{1},P_{2}^{j},\gamma )<\lambda \right\}}w\left(j\right)+\sum_{\left\{j\in U:\;\mathrm{GJS}(P_{1},P_{2}^{j},\gamma )=\lambda \right\}}\Phi \left(r\right)w\left(j\right). \end{array}$$

In view of (A35), Equation (A41) indicates that

(A42) $$\begin{array}{c} r\leq \sup\left\{r\;|\sum_{\left\{j\in U:\;\mathrm{GJS}(P_{1},P_{2}^{j},\gamma )<\lambda \right\}}w\left(j\right)+\sum_{\left\{j\in U:\;\mathrm{GJS}(P_{1},P_{2}^{j},\gamma )=\lambda \right\}}\Phi_{j}\left(r\right)w\left(j\right)\leq \epsilon \right\}. \end{array}$$

□

## Appendix F

**Proof** **of** **Equation (A28)**). We shall show

(A43) $$\begin{array}{cc} \; & \frac{1}{n}\sum_{i=1}^{N}\iota_{1}\left(t_{1,i}|P_{1},P_{2}^{j},\gamma \right)+\frac{1}{n}\sum_{i=1}^{n}\iota_{2}\left(x_{i}|P_{1},P_{2}^{j},\gamma \right)\leq \lambda +\frac{r}{\sqrt{n}}+O\left(\frac{\log n}{n}\right)\\ \; & =\frac{1}{n}\sum_{i=1}^{N}\left\{\iota_{1}\left(t_{1,i}|P_{1},P_{2}^{j},\gamma \right)-E_{P_{1}}\left[\iota_{1}\left(X|P_{1},P_{2}^{j},\gamma \right)\right]\right\}+\frac{1}{n}\sum_{i=1}^{n}\{\iota_{2}\left(x_{i}|P_{1},P_{2}^{j},\gamma \right)\\ \; & \;\;\;\;\;\;\;\;\;\;\;\;-E_{P_{2}}\left[\iota_{2}\left(X|P_{1},P_{2}^{j},\gamma \right)\right]\}\leq \lambda +\frac{r}{\sqrt{n}}-\mathrm{GJS}\left(P_{1},P_{2}^{j},\gamma \right)+O\left(\frac{\log n}{n}\right). \end{array}$$

The left-hand side of (A43) can be rewritten as follows:

(A44) $$\begin{array}{cc} \; & \frac{1}{n}\sum_{i=1}^{N}\iota_{1}\left(t_{1,i}|P_{1},P_{2}^{j},\gamma \right)+\frac{1}{n}\sum_{i=1}^{n}\iota_{2}\left(x_{i}|P_{1},P_{2}^{j},\gamma \right)\leq \lambda +\frac{r}{\sqrt{n}}+O\left(\frac{\log n}{n}\right)\\ ⟺ & \frac{1}{n}\sum_{i=1}^{N}\iota_{1}\left(t_{1,i}|P_{1},P_{2}^{j},\gamma \right)-\gamma E_{P_{1}}\left[\iota_{1}\left(X|P_{1},P_{2}^{j},\gamma \right)\right]+\frac{1}{n}\sum_{i=1}^{n}\iota_{2}\left(x_{i}|P_{1},P_{2}^{j},\gamma \right)\\ \; & \;\;\;\;\;\;\;\;\;\;\;\;-E_{P_{2}}\left[\iota_{2}\left(X|P_{1},P_{2},\gamma \right)\right]\leq \lambda +\frac{r}{\sqrt{n}}-\mathrm{GJS}\left(P_{1},P_{2}^{j},\gamma \right)+O\left(\frac{\log n}{n}\right)\\ ⟺ & \frac{1}{n}\sum_{i=1}^{N}\iota_{1}\left(t_{1,i}|P_{1},P_{2}^{j},\gamma \right)-\frac{1}{n}\sum_{i=1}^{N}E_{P_{1}}\left[\iota_{1}\left(X|P_{1},P_{2}^{j},\gamma \right)\right]+\frac{1}{n}\sum_{i=1}^{n}\iota_{2}\left(x_{i}|P_{1},P_{2}^{j},\gamma \right) \end{array}$$

(A45) $$\begin{array}{cc} \; & \;\;\;\;\;\;\;\;\;\;\;\;-\frac{1}{n}\sum_{i=1}^{n}E_{P_{2}}\left[\iota_{2}\left(X|P_{1},P_{2},\gamma \right)\right]\leq \lambda +\frac{r}{\sqrt{n}}-\mathrm{GJS}\left(P_{1},P_{2}^{j},\gamma \right)+O\left(\frac{\log n}{n}\right), \end{array}$$

where (A44) follows from (47). Therefore, we obtain Equation (A28). □
